# SIRT2 deletion enhances KRAS-induced tumorigenesis *in vivo* by regulating K147 acetylation status

**DOI:** 10.18632/oncotarget.12015

**Published:** 2016-09-13

**Authors:** Ha Yong Song, Marco Biancucci, Hong-Jun Kang, Carol O'Callaghan, Seong-Hoon Park, Daniel R. Principe, Haiyan Jiang, Yufan Yan, Karla Fullner Satchell, Kirtee Raparia, David Gius, Athanassios Vassilopoulos

**Affiliations:** ^1^ Department of Radiation Oncology, Laboratory for Molecular Cancer Biology, Robert H. Lurie Comprehensive Cancer Center, Feinberg School of Medicine, Northwestern University, Chicago, IL, USA; ^2^ Department of Microbiology-Immunology, Feinberg School of Medicine, Northwestern University, Chicago, IL, USA; ^3^ Department of Radiation Oncology, Robert H. Lurie Comprehensive Cancer Center, Feinberg School of Medicine, Northwestern University, Chicago, IL, USA; ^4^ Department of Pathology, Northwestern University, Feinberg School of Medicine, Chicago, IL, USA

**Keywords:** KRAS, SIRT2, acetylation, lung cancer, pancreas transformation

## Abstract

The observation that cellular transformation depends on breaching a crucial KRAS activity threshold, along with the finding that only a small percentage of cellsharboring *KRAS* mutations are transformed, support the idea that additional, not fully uncovered, regulatory mechanisms may contribute to KRAS activation. Here we report that Kras^G12D^ mice lacking *Sirt2* show an aggressive tumorigenic phenotype as compared to Kras^G12D^ mice. This phenotype includes increased proliferation, KRAS acetylation, and activation of RAS downstream signaling markers. Mechanistically, KRAS K147 is identified as a novel SIRT2-specific deacetylation target by mass spectrometry, whereas its acetylation status directly regulates KRAS activity, ultimately exerting an impact on cellular behavior as revealed by cell proliferation, colony formation, and tumor growth. Given the significance of KRAS activity as a driver in tumorigenesis, identification of K147 acetylation as a novel post-translational modification directed by SIRT2 *in vivo* may provide a better understanding of the mechanistic link regarding the crosstalk between non-genetic and genetic factors in KRAS driven tumors.

## INTRODUCTION

Sirtuin genes are the human and murine homologs of the *S. cerevisiae* Sir2 gene that have been shown to regulate both replicative and overall lifespan [1]. While the precise role of sirtuins in mammalian lifespan regulation is yet to be fully defined, it was recently demonstrated that male transgenic mice overexpressing *SIRT6* live longer than their wild-type littermates [2]. Despite the observed discrepancies and the scarcity of detailed mechanisms regarding the role of sirtuins in longevity, it is well established that they do appear to direct critical acetylome signaling networks responding to caloric restriction (CR) [3], and following stress, several mice lacking one of the sirtuin genes develop illnesses that mimic those observed in humans that are strongly connected to increasing age [4]. Consistent with this, mice lacking *Sirt2* [5] develop multiple epithelial malignancies, including pancreatic ductal adenocarcinoma (PDAC) and lung adenocarcinoma (LACA). Based on these findings, it has been suggested that sirtuins are energy/nutrient stress sensor proteins that alter the activity of downstream signaling networks and targets via post-translational modifications (PTMs) involving lysine deacetylation in response to specific types of cellular stress.

It is well established that *KRAS* mutations are observed in 95% of patients with PDAC [6, 7] and roughly 30% of LACA cases [7]. However, similar to most human malignancies, it is also clear that additional aberrant genetic and/or biochemical events are ultimately required for carcinogenesis. Interestingly, healthy people have cells expressing oncogenic *KRAS* in different organs, including the pancreas, colon, and lungs, at rates far exceeding the rates of cancer development [8, 9]. In addition, whereas the number of cells that ultimately are transformed is only a small fraction of those expressing mutant *Kras* (*mtKras*) in mouse models [10], there is emerging evidence suggesting that KRAS activity is increased in cells derived from *mtKras*-driven PDAC as compared to non-transformed pancreas expressing *mtKras* [11]. Therefore, identification of mechanisms which may contribute to breaching a crucial enzymatic KRAS activity threshold to initiate carcinogenesis, even in the presence of activating KRAS mutations, may fill the critical gap in knowledge related to KRAS-driven tumorigenesis.

Based on our previous finding that *Sirt2* *^-/-^* mice develop spontaneous tumors, and a recent study showing that KRAS is acetylated in cancer cells [12], we aimed to determine the role of SIRT2 in KRAS-induced tumorigenesis *in vivo*. Here we report that Sirt2^-/-^-Kras^G12D^ mice show enhanced pancreas transformation as well as lung tumorigenesis as compared to Kras^G12D^ mice. These phenotypes are associated with increased proliferation and KRAS acetylation as well as downstream RAS-activated signaling markers, suggesting carcinogenesis is more aggressive when *Sirt2* is deleted. These results can be explained by the finding that the acetylation status of K147 directed by SIRT2 regulates KRAS's GTP-bound “active state,” as shown by using mutants that mimic either acetylation (K147Q) or deacetylation (K147R). The effect of K147 acetylation on KRAS activity impacts both the transformative and oncogenic properties of KRAS. These results, together with the fact that K147 acetylation can be detected in tissues *in vivo*, highlight the role of this reversible PTM in regulating KRAS activity, and, more importantly, identify for the first time K147 acetylation as an oncogenic modification directed by SIRT2.

## RESULTS

### Deletion of Sirt2 induces KRAS-mediated pancreas transformation

**Figure 1:**
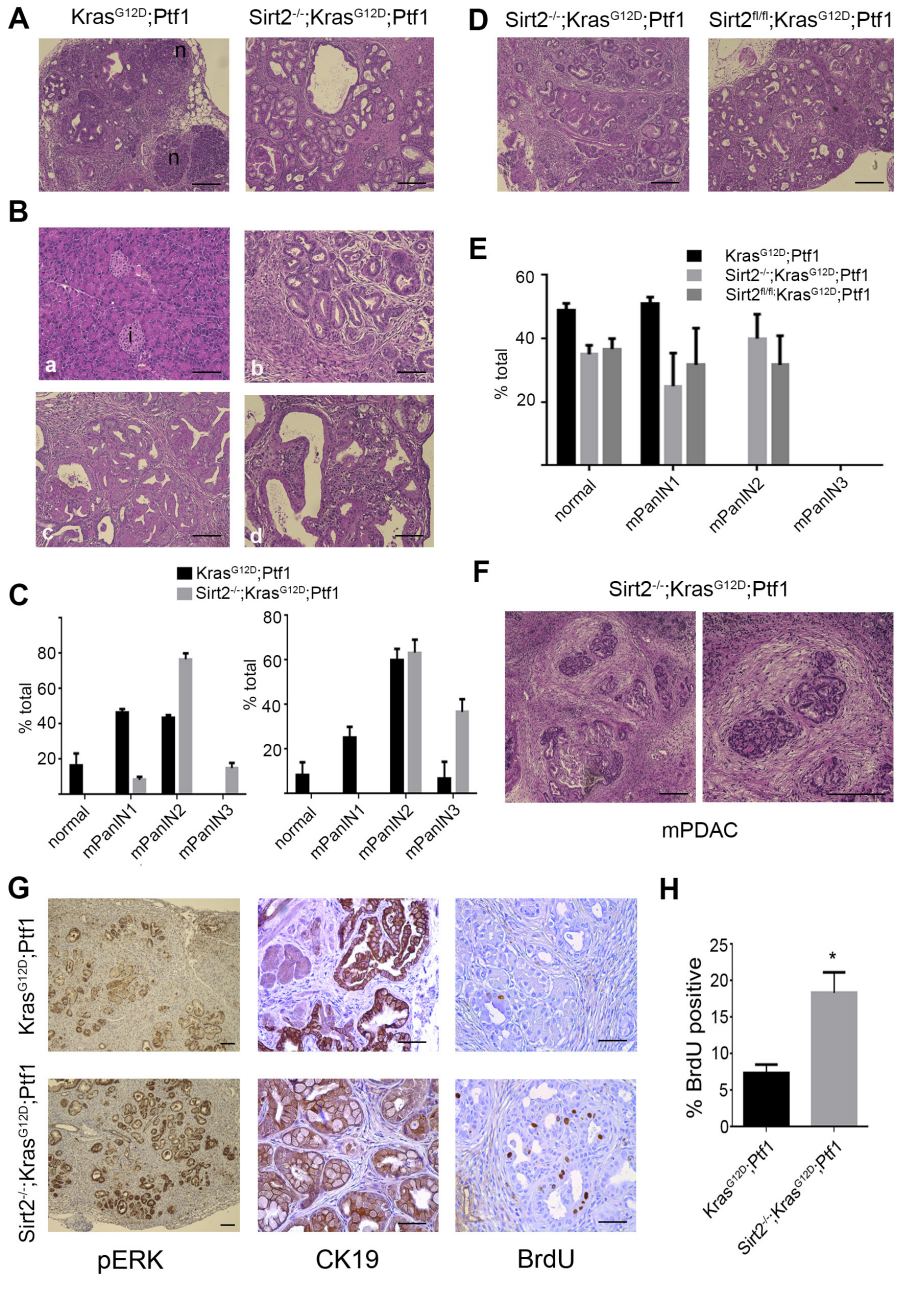
Mice lacking *Sirt2* and expressing *mtKras* exhibit enhanced development of PanIN and progression to PDAC **A.** Pancreata from Kras^G12D^-Ptf1 and Sirt2^-/-^-Kras^G12D^-Ptf1 mice were harvested at 4 months of age (*n* = 5-8), and samples were fixed and analyzed by H&E staining for pancreas morphology. A representative image from each genotype is shown. Normal acinar tissue (n) is found in Kras^G12D^-Ptf1 mice but not in Sirt2^-/-^-Kras^G12D^-Ptf1 mice. Scale bar 200 μM (10x). **B.** Histology of the pancreas and different grades of lesions found in the Sirt2^-/-^-Kras^G12D^-Ptf1 mice. Representative images are shown: (a) normal tissue with both acinar cells and islets (i), (b) PanIN1, (c) PanIN2; and (d) PanIN3. Scale bar 200 μM (10x). **C.** Quantification of lesions found at 4 months (left panel) and 6 months (right panel) of age is presented. Data represent mean ± SEM. **D.** Pancreata from Sirt2^-/-^-Kras^G12D^-Ptf1 (*n* = 5-8) and Sirt2^fl/fl^-Kras^G12D^-Ptf1 (*n* = 4) mice were harvested at 2 months of age, and samples were fixed and analyzed by H&E staining for pancreas morphology. A representative image from lesions found in each genotype is shown. Scale bar 200 μM (10x). **E.** Quantification of normal tissue and lesions found at 2 months of age in Kras^G12D^-Ptf1, Sirt2^-/-^-Kras^G12D^-Ptf1, and Sirt2^-/-^-Kras^G12D^-Ptf1 mice is presented. Data represent mean ± SEM. **F.** Pancreata from the Sirt2^-/-^-Kras^G12D^-Ptf1 mice that displayed mouse PDAC (mPDAC) were H&E stained, and a representative image of PDAC is shown. Magnification (20x vs 10x left) of the mPDAC area is shown in the right panel. Scale bar 200 μM. **G.** Pancreata from Kras^G12D^-Ptf1 and Sirt2^-/-^ -Kras^G12D^-Ptf1 mice were isolated, and sections were subsequently IHC stained with antibodies against pERK (left, 5x, scale bar 200 μM), CK19 (middle, 20x, scale bar 100 μM) and BrdU (right, 20x, scale bar 100 μM). **H.** Quantification of BrdU-positive cells found in pancreas of Kras^G12D^-Ptf1 and Sirt2^-/-^-Kras^G12D^-Ptf1 mice. Data represent mean ± SD (*n* = 3), *p < 0.05.

To determine the role of SIRT2 in KRAS-induced pancreas transformation, the pancreatic epithelium-specific *Kras^G12D^* mice, which were generated using the well-established *LSL-Kras^G12D^* knock-in mouse model [13] and the *Ptf1^Cre^* driver line [14] to direct recombination in pancreas, were crossed with *Sirt2^-/-^* mice [5] to generate *Sirt2^+/+^;LSL-Kras^G12D^;Ptf1^Cre^* and *Sirt2^-/-^;LSL-Kras^G12D^;Ptf1^Cre^* mice (referred to here as Kras^G12D^-Ptf1 and Sirt2^-/-^-Kras^G12D^-Ptf1 mice, respectively). At 4 months of age, the Sirt2^-/-^-Kras^G12D^-Ptf1 mice exhibited a complete loss of normal glandular architecture, with no discernible normal tissue (Figure 1A right, see absence of normal tissue (n)). This abnormal architecture was accompanied by severe fibrosis (Figure S1A, right panel) as observed by trichrome staining, especially around areas with detectable pancreatic lesions. Histopathologic analyses revealed that all mice developed pancreatic intraepithelial neoplasia (PanIN) of different grades (Figure 1B). However, Sirt2^-/-^-Kras^G12D^-Ptf1 mice showed an increase in induction and PanIN progression compared to Kras^G12D^-Ptf1 mice as revealed by the amount and grade of these lesions both at 4 and 6 months of age (Figure 1C). This is consistent with a tumor suppressor role of *SIRT2* in the pancreas, which is also in line with lower *SIRT2* mRNA levels detected in pancreatic adenocarcinoma compared to normal tissue (Figure S1B).

To explore whether the *Kras*-induced pancreas transformation found in *Sirt2^-/-^* mice is due to a cell autonomous mechanism, we generated *Sirt2* conditional knockout (*Sirt2^fl/fl^*) mice (Figure S1C-E) that were crossed with the *LSL-Kras^G12D^;Ptf1^Cre^* mouse model to study the effect of pancreatic epithelium-specific *Sirt2* deletion in the *Kras^G12D^* knock-in mouse model. Histopathologic analyses showed similar patterns of induction and PanIN progression in both the Sirt2^fl/fl^-Kras^G12D^-Ptf1 and Sirt2^-/-^-Kras^G12D^-Ptf1 mice (Figure 1D, 1E), suggesting that SIRT2 acts autonomously in pancreatic cells. Of note, some Sirt2^-/-^-Kras^G12D^-Ptf1 mice (two out of ten) developed areas of PDAC at 8 months of age (Figure 1F), an acceleration of the timeframe of PDAC development in Kras^G12D^-Ptf1 mice (≥1 year). In addition, these mice contained areas of tumor cells that appeared disorganized and were surrounded by dense fibrosis, as evidenced by a robust desmoplastic response (Figure 1F).

Oncogenic KRAS activates a plethora of signaling pathways, including the canonical Raf/MEK/ERK pathway. Lesions from both Kras^G12D^-Ptf1 and Sirt2^-/-^ -Kras^G12D^-Ptf1 mice that were confirmed to be of pancreatic origin after positive staining with CK19, were also stained for phosphorylated ERK (pERK) as an indirect marker of KRAS activity in these mice (Figure 1G). Sirt2^-/-^-Kras^G12D^-Ptf1 pancreas showed higher pERK levels compared to Kras^G12D^-Ptf1 mice, indicating that deletion of *Sirt2* is associated with increased downstream KRAS signaling. Finally, proliferation analysis using BrdU labeling showed active proliferation in Sirt2^-/-^-Kras^G12D^-Ptf1 pancreas as compared to Kras^G12D^-Ptf1 pancreas (Figure 1G, 1H). All these data together further indicate that loss of *Sirt2* induces KRAS-mediated pancreas transformation.

### Deletion of Sirt2 increases KRAS-induced lung adenocarcinoma

**Figure 2:**
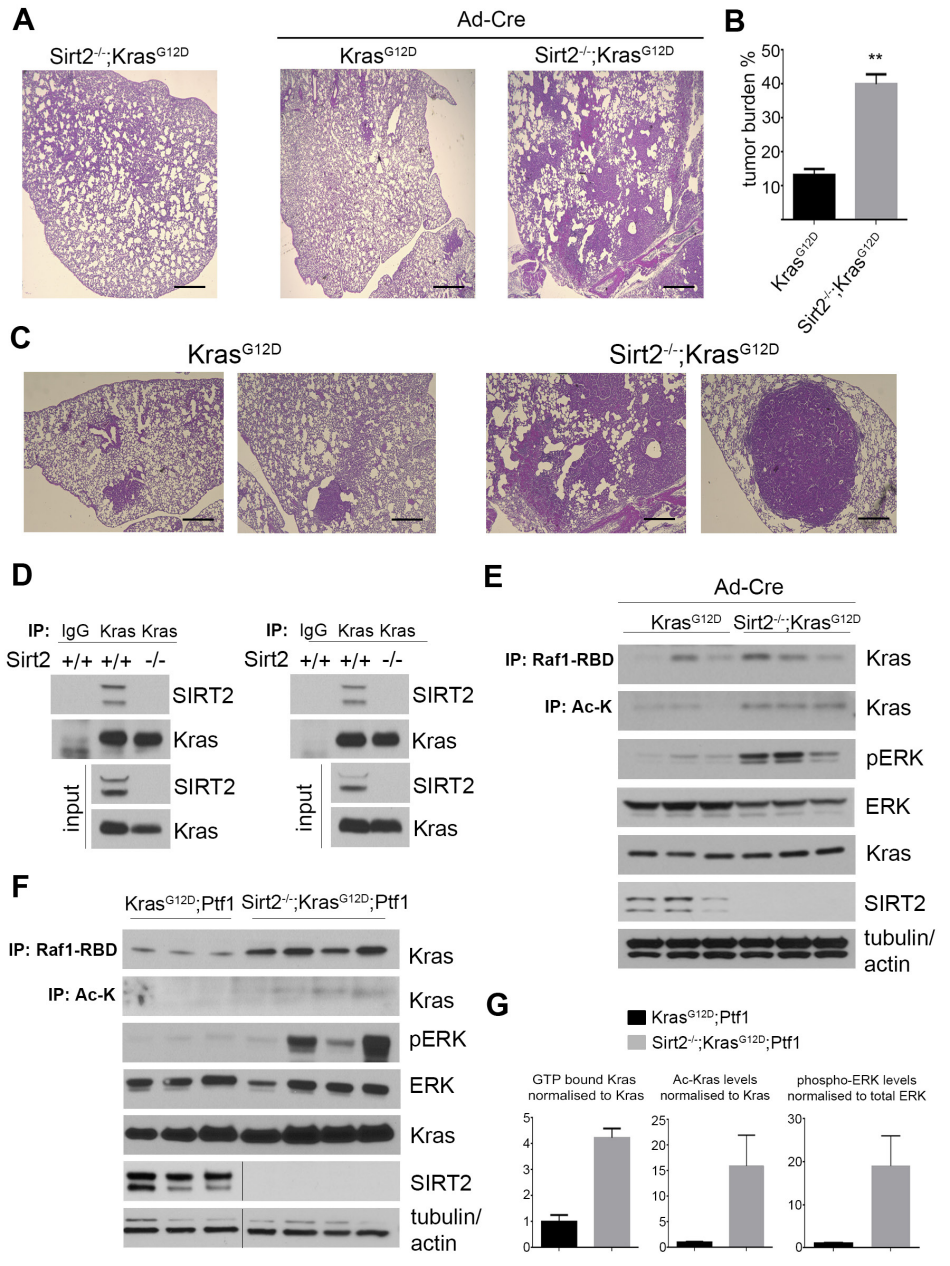
Loss of *Sirt2* enhances KRAS^**G12D**^-induced lung adenocarcinoma and KRAS acetylation is associated with increased activity *in vivo* **A.**-**C.** The lungs from Kras^G12D^ and Sirt2^-/-^-Kras^G12D^ mice (*n* = 5 for each genotype), four months after intranasal administration of adenoCRE (Ad-Cre), were harvested, fixed, sectioned, and H&E stained. **A.** Representative images from lungs (2.5x) of control Sirt2^-/-^-Kras^G12D^ mice (untreated with adenoCRE), and lung tumors developed in Kras^G12D^ and Sirt2^-/-^-Kras^G12D^ mice are shown. Scale bar 50 μM. **B.** Tumor burden at 4 months in lungs from Kras^G12D^ and Sirt2^-/-^ -Kras^G12D^ mice is presented. Data represent mean ± SEM, **p < 0.01. **C.** Higher magnification of lung histology in both Kras^G12D^ (left, 10x) and Sirt2^-/-^-Kras^G12D^ (right, 10x) mice is shown. Scale bar 200 μM. **D.** Endogenous KRAS was immunoprecipitated from lysates of either lung (left) or pancreas (right) tissues. Interaction was confirmed by western blotting using anti-SIRT2 and anti-KRAS antibodies. Endogenous levels of both KRAS and SIRT2 are shown as input. **E.** The lungs from Kras^G12D^ and Sirt2^-/-^-Kras^G12D^ mice, 2 months after intranasal administration of adenoCRE, were harvested and analyzed for KRAS acetylation and KRAS activity. KRAS acetylation was detected by immunoprecipitation with a pan anti-Ac-K antibody, and KRAS activity was detected by immunoprecipitation with Raf1-RBD and by blotting for pERK. ERK, KRAS, and SIRT2 inputs are shown as controls, and actin and tubulin were used as loading controls. **F.** The pancreata from Kras^G12D^-Ptf1 and Sirt2^-/-^-Kras^G12D^-Ptf1 mice were harvested and analyzed for KRAS activity and KRAS acetylation as described in panel (E). **G.** Quantification of KRAS activity, KRAS acetylation levels, and phosphorylation levels of ERK from panel (F). Data represent mean ± SEM.

To determine the role of SIRT2 in KRAS-dependent lung adenocarcinoma, the well-established *LSL-Kras^G12D^* knock-in mouse model was crossed with *Sirt2^-/-^* mice to generate *Sirt2^+/+^;LSL-Kras^G12D^* and *Sirt2^-/-^;LSL-Kras^G12D^* mice (referred to here as Kras^G12D^ and Sirt2^-/-^-Kras^G12D^ mice, respectively) followed by intranasal administration of adenoviral *Cre* (adenoCRE) to induce *mtKras* expression in the lungs [15]. Examination of the lungs in a cohort of untreated Sirt2^-/-^-Kras^G12D^ mice as well as Kras^G12D^ and Sirt2^-/-^-Kras^G12D^ mice 4 months after exposure to adenoCRE revealed that *Sirt2*-null mice had a significantly greater number of tumor lesions in the lungs (Figure 2A, 2B), as well as larger tumor area (Figure 2C), than Kras^G12D^ mice.

Histopathologic examination of lungs at an earlier time point after adenoCRE administration showed similar results. In particular, 2 months after adenoCRE administration, the Sirt2^-/-^-Kras^G12D^ mice lungs exhibited increased numbers of tumor cell nests per 10x field (Figure S1F) and increased tumor size (Figure S1G). Histopathological analyses showed that Sirt2^-/-^-Kras^G12D^ mice clearly developed lung tumors (Figure S1H, two right panels), as compared to mostly scattered areas of atypical adenomatous hyperplasia (AAH) developed in the Kras^G12D^ mice (Figure S1H, two left panels). The tumors were adenocarcinomas, as determined by thyroid transcription factor-1 staining (data not shown). Furthermore, Sirt2^-/-^-Kras^G12D^ serial lung sections showed nests of tumor cells exhibiting increased *in vivo* pERK (Figure S1I, middle panels) and Ki-67 staining (Figure S1I, right panels), implying increased KRAS activity and tumorigenicity in the lungs when *Sirt2* is deleted. Collectively, these results suggest that *Sirt2* deletion enhances the progression of oncogenic KRAS-induced lung adenocarcinomas.

### Increased acetylation of KRAS due to Sirt2 loss is associated with increased KRAS activity

To start unraveling the mechanistic details regarding either the direct or indirect effect of SIRT2 on KRAS, we checked whether there is a direct interaction between the two proteins. Of note, we detected interaction of the two proteins in both pancreas and lung (Figure 2D) tissues found to exhibit enhanced KRAS-mediated transformation and/or tumorigenicity when *Sirt2* was lost, as presented earlier. The presence of both proteins in the same complex was further confirmed in reciprocal co-immunoprecipitation experiments after overexpressing both KRAS and SIRT2 in 293T cells (data not shown), as well as after checking endogenous proteins in *Sirt2^+/+^* primary MEFs (Figure S2A). Furthermore, KRAS was detected to specifically interact with SIRT2 but not other members of the sirtuin family (Figure S2B, C) arguing for a direct regulatory function of SIRT2 on KRAS. More importantly, tissues depleted of *Sirt2* exhibited a significant increase in KRAS acetylated levels which was further associated with increased KRAS activity (Figure 2E-2G). In particular, GTP-bound “active” KRAS, detected through a specific protein interaction with the Raf1 Ras-binding domain (Raf1-RBD), was enriched in the *Sirt2^-/-^* tissues. Under the same experimental conditions, enhanced KRAS activity was associated with increased pERK levels (Figure 2E-2G), further establishing the positive effect of KRAS acetylation on its activity *in vivo*. Taken together, these results favor the scenario of KRAS being a SIRT2 deacetylation target *in vivo*. The generality of the regulatory role of SIRT2 in KRAS signaling was also confirmed in *Sirt2^-/-^* MEFs (Figure S2D). In addition, in HCT116 cells, which express a wild-type and a mutant *Kras* allele, infection with lenti-sh*SIRT2* increased both KRAS activity, as evidenced by increased pERK levels (Figure S2E), and colony formation, as revealed by enhanced growth in soft agar (Figure S2F, G).

To establish KRAS as a legitimate SIRT2 deacetylation target, a series of cell culture experiments were performed. To assess whether SIRT2 deacetylates KRAS, 293T cells were co-transfected either with *HA-KRAS* or *HA-KRAS^G12V^*, with wild-type *Flag-SIRT2*, as well as with p300/CBP based on our results showing that these two are the main histone acetyl transferases (HATs) to acetylate KRAS (Figure S2H). Following immunoprecipitation with an anti-acetyl-lysine (Ac-K) antibody, both wild-type and KRAS^G12V^ were deacetylated by SIRT2 (Figure S2I, lane 3 vs lane 2 and lane 6 vs lane 5, respectively). Increased KRAS activity upon treatment with both nicotinamide, an inhibitor of all members of the sirtuin family, and AGK2, a specific SIRT2 inhibitor (Figure S2J), highlights the unique role of SIRT2 among other sirtuins as a regulator of KRAS activity. Finally, KRAS activity was decreased in co-transfection experiments using wild-type SIRT2 (*SIRT2^wt^*), but not a SIRT2 deacetylation null mutant gene (*SIRT2^dn^*) (Figure S2K), suggesting that SIRT2 regulates KRAS activity through its deacetylation activity. Interestingly, the same effect on KRAS^G12V^ activity was observed (data not shown), implying that both wild-type and mutant KRAS can be regulated through SIRT2-mediated deacetylation. Together these results demonstrate that KRAS contains a reversible acetyl-lysine and aberrant regulation of KRAS when *SIRT2* is lost may result in induced KRAS activity.

### Acetylation status of K147 directs KRAS activity and transformative properties

To identify specific SIRT2 target lysines, lysates from 293T cells expressing either *Flag-KRAS^G12V^* alone (sample 1) or *Flag-KRAS^G12V^* and *SIRT2* (sample 2) were run on a gel and bands corresponding to the molecular weight of Flag-KRAS were excised and analyzed by mass spectrometry (Figure S3A-C). In the absence of SIRT2 (Figure S3A, sample 1), acetylated lysines 104 and 147 (K104, K147 in red circles) were detected with the peptides identified under this condition (highlighted in yellow). When *SIRT2* was overexpressed (Figure S3A, sample 2), K147 acetylation was undetectable in contrast to K104 acetylation, implying that K147 is a SIRT2-specific reversibly acetylated lysine target.

**Figure 3:**
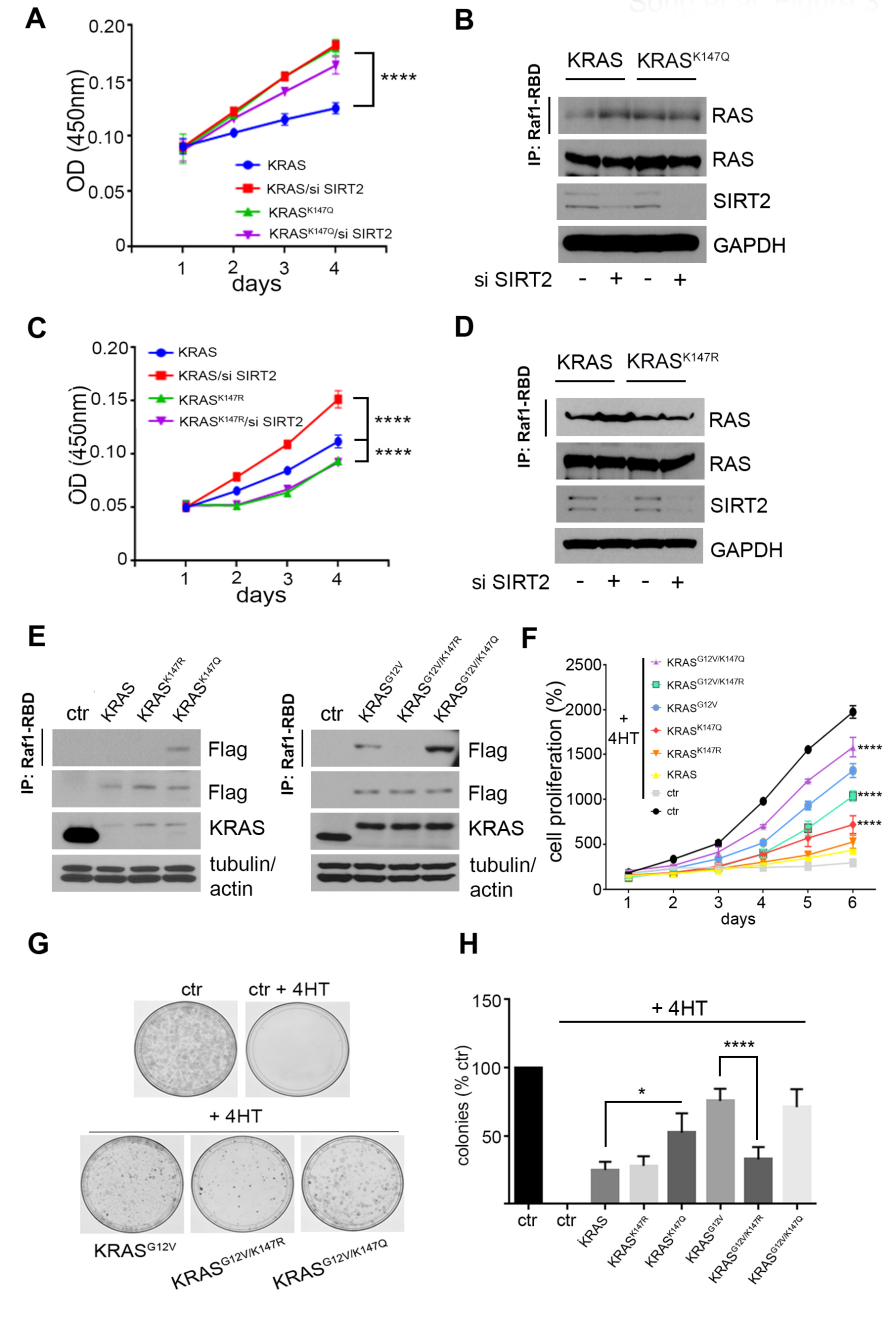
K147 acetylation status directed by SIRT2 regulates KRAS dependent proliferation **A.** *Kras* MEFs were infected with lenti-*KRAS* or lenti-*KRAS*, and subsequently treated with 4-hydroxytamoxifen (4HT) to delete endogenous Kras. Next, *Sirt2* was knocked down by siRNA and cell proliferation was monitored. Data represent mean ± SD of three independent experiments, ****p < 0.0001. **B.** KRAS activity in lysates from cells used in (A) was detected by immunoprecipitation of GTP-bound KRAS using Raf1-RBD agarose beads followed by immunoblotting using an anti-RAS antibody. KRAS, SIRT2 and GAPDH levels were checked by immunoblotting as shown. **C.**, **D.** *Kras* MEFs were infected with lenti-*KRAS* or lenti-*KRAS* and same experiments as described in (A, B) were performed. For cell proliferation, data represent mean ± SD of three independent experiments, ****p < 0.0001. **E.** *Kras* MEFs were infected with lenti-*KRAS*, lenti-*KRAS*, lenti-*KRAS*(left), as well as lenti-*KRAS*, lenti-*KRAS*, and lenti-*KRAS* (right) and subsequently treated with 4-hydroxytamoxifen (4HT) to delete endogenous Kras. Active KRAS was detected by immunoprecipitation of GTP-bound KRAS using Raf1-RBD agarose beads followed by immunoblotting using an anti-Flag antibody. Levels of exogenously expressed KRAS proteins are shown in whole cell lysates by western blotting using an anti-Flag antibody. Actin and tubulin are used as loading controls. **F.**, **G.** The same cells as in (E) were used to check proliferation rate by measuring the number of cells for 6 consecutive days (F) and by determining the colony formation ability after 21 days (G). For cell proliferation, data represent mean ± SD of three independent experiments, ****p < 0.0001 *KRAS*and *KRAS*vs *KRAS*MEFs, ****p < 0.0001 *KRAS*vs *KRAS* MEFs. **H.** Quantification of data in panel (G) is shown. Data represent the mean ± SEM of three independent experiments, *p < 0.05 *KRAS*vs *KRAS* MEFs, ****p < 0.0001 *KRAS*vs *KRAS*MEFs.

It has been previously shown that substitution of a lysine with a glutamine mimics the acetylated state, while substitution with an arginine mimics deacetylation [16]. For this, we generated *K147Q* and *K147R KRAS* mutants to determine the effect of K147 acetylation on KRAS activity. The KRAS mutants were expressed in *Kras^lox^* MEFs (*Hras^-/-^;Nras^-/-^;Kras^lox/lox^;RERTn^ert/ert^*) where the conditional *Kras^lox^* alleles are fully excised upon expression of the *Cre* recombinase, inducible in the presence of 4-hydroxytamoxifen (4HT), resulting in *Rasless* MEFs [17]. In particular, *Kras^lox^* MEFs were infected with lenti-*KRAS*, lenti-*KRAS^K147R^*, lenti-*KRAS^K147Q^*, lenti-*KRAS^G12V^*, lenti-*KRAS^G12V-K147R^* and lenti-*KRAS^G12V-K147Q^* and subsequently treated with 4HT, resulting in cells expressing only the lentiviral constructs of *KRAS* and *KRAS^G12V^*. Cell proliferation rate was significantly higher in MEFs expressing *KRAS* after knocking down *Sirt2* consistent with increased KRAS activity detected in these cells, whereas decreased *Sirt2* expression didn't affect proliferation in MEFs expressing either *KRAS^K147R^* or *KRAS^K147Q^* (Figure 3A-3D). Next we examined the impact of K147 acetylation status in both wild-type KRAS and mutant KRAS^G12V^. MEFs expressing *KRAS^K147Q^* exhibited increased amounts of “active” GTP bound KRAS, as measured by the Ras pulldown assay, compared to the *KRAS* and *KRAS^K147R^* infected cells (Figure 3E, left), suggesting that acetylation enhances wild-type KRAS activity. In cells expressing *KRAS^G12V^*, expression of *KRAS^G12V-K147Q^* produced an increase in the amount of active protein compared to KRAS^G12V^, whereas less active protein was observed with expression of *KRAS^G12V-K147R^*. (Figure 3E, right). The effect of K147 acetylation status on KRAS activity was confirmed in 293T cells after transient overexpression of the *KRAS* and *KRAS^G12V^* acetylation mutants (data not shown). MEFs expressing *KRAS^K147Q^* exhibited increased proliferation as determined by measuring the number of cells and colonies (Figure 3F-3H), compared to cells expressing either *KRAS* or *KRAS^K147R^*. In *KRAS^G12V^* expressing MEFs, KRAS^G12V-K147Q^ increased the proliferation rate, whereas KRAS^G12V-K147R^ exerted the opposite effect (Figure 3F-3H).

**Figure 4:**
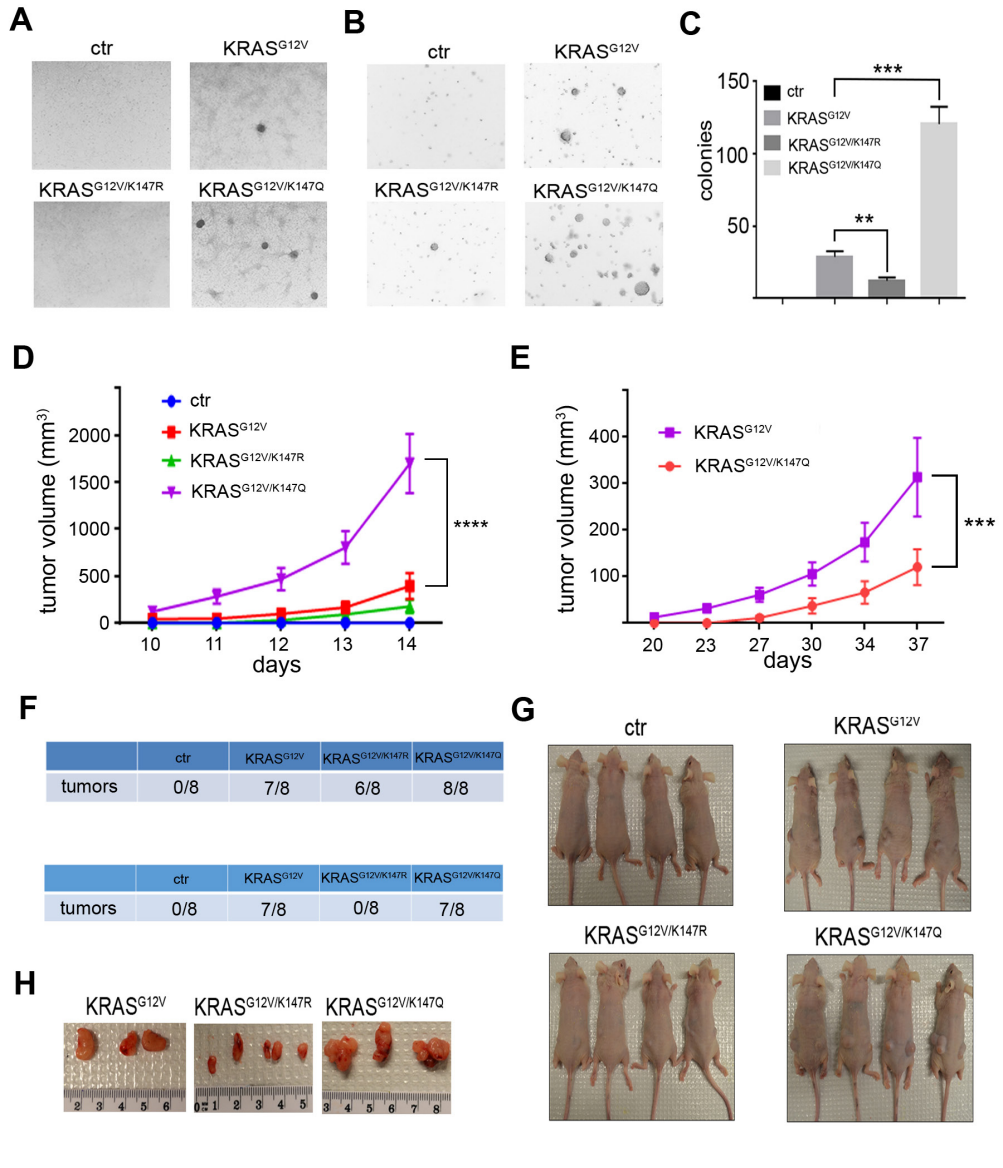
K147 acetylation status regulates KRAS transformative properties **A.**, **B.** Transforming activity of NIH3T3 cells expressing *KRAS*, *KRAS* and *KRAS* after knocking down endogenous *Kras* was checked by testing colonies formed when cells were grown under confluency (A) and in soft agar (B). **C.** Quantification of data in panel (B) is shown. Data represent the mean ± SD of three independent experiments, **p < 0.01 *KRAS*vs *KRAS* cells, ***p < 0.001 *KRAS*vs *KRAS* cells. **D.** NIH3T3 cells expressing *KRAS*, *KRAS* and *KRAS* after knocking down endogenous *Kras* were injected subcutaneously into nude mice, and tumor growth was monitored by measuring tumor volume (*n* = 8 tumor injections were performed for each cell line). Data represent mean ± SEM, ****p < 0.0001 *KRAS*vs *KRAS* tumors. **E.** *Kras* MEFs infected with lenti-*KRAS*, lenti-*KRAS* and lenti-*KRAS* followed by treatment with 4HT were injected subcutaneously into nude mice, and tumor growth was monitored by measuring tumor volume (*n* = 8 tumor injections were performed for each cell line). Data represent mean ± SEM, ***p < 0.001 *KRAS*vs *KRAS* tumors. **F.** Number of tumors formed in nude mice injected with NIH3T3 cells expressing *KRAS*, *KRAS* and *KRAS* after knocking down endogenous *Kras* (upper) and *Kras* MEFs infected with lenti-*KRAS*, lenti-*KRAS*, and lenti-*KRAS* followed by treatment with 4HT (lower). **G.** Characteristic images of mice bearing the subcutaneous tumors after injecting the different cells. **H.** Subcutaneous tumors were removed after sacrificing the mice, and characteristic images of the tumors are shown.

To determine the impact of K147 acetylation status on KRAS transformative properties, NIH3T3 cells expressing *KRAS^G12V^* acetylation mutants after knocking down endogenous *Kras* were constructed. Of note, NIH3T3 cells expressing *KRAS^G12V-K147Q^* exhibited increased proliferation (Figure S4A-C), consistent with previous results in MEFs, excluding the possibility that the observed differences in proliferation could be attributed either to the different integration sites after viral infection or to a cell type-specific phenomenon. More importantly, these cells formed more colonies when grown both under confluence and in soft agar (Figure 4A-4C), indicating enhanced transforming activity of KRAS upon K147 acetylation. To further establish that K147 acetylation is an oncogenic PTM, both NIH3T3 cells and *Rasless* MEFs expressing the different *KRAS^G12V^* acetylation mutants were used for subcutaneous injections into nude mice, and tumor growth was monitored. In accordance with the cell culture results, *KRAS^G12V-K147Q^-*expressing cells exhibited a significant increase in tumor growth rate relative to cells expressing *KRAS^G12V^*, as evidenced by the increased volume of tumors developed in these mice (Figure 4D-4H and Figure S4D, E).

### K147 acetylation alters the nucleotide exchange kinetics of KRAS

**Figure 5:**
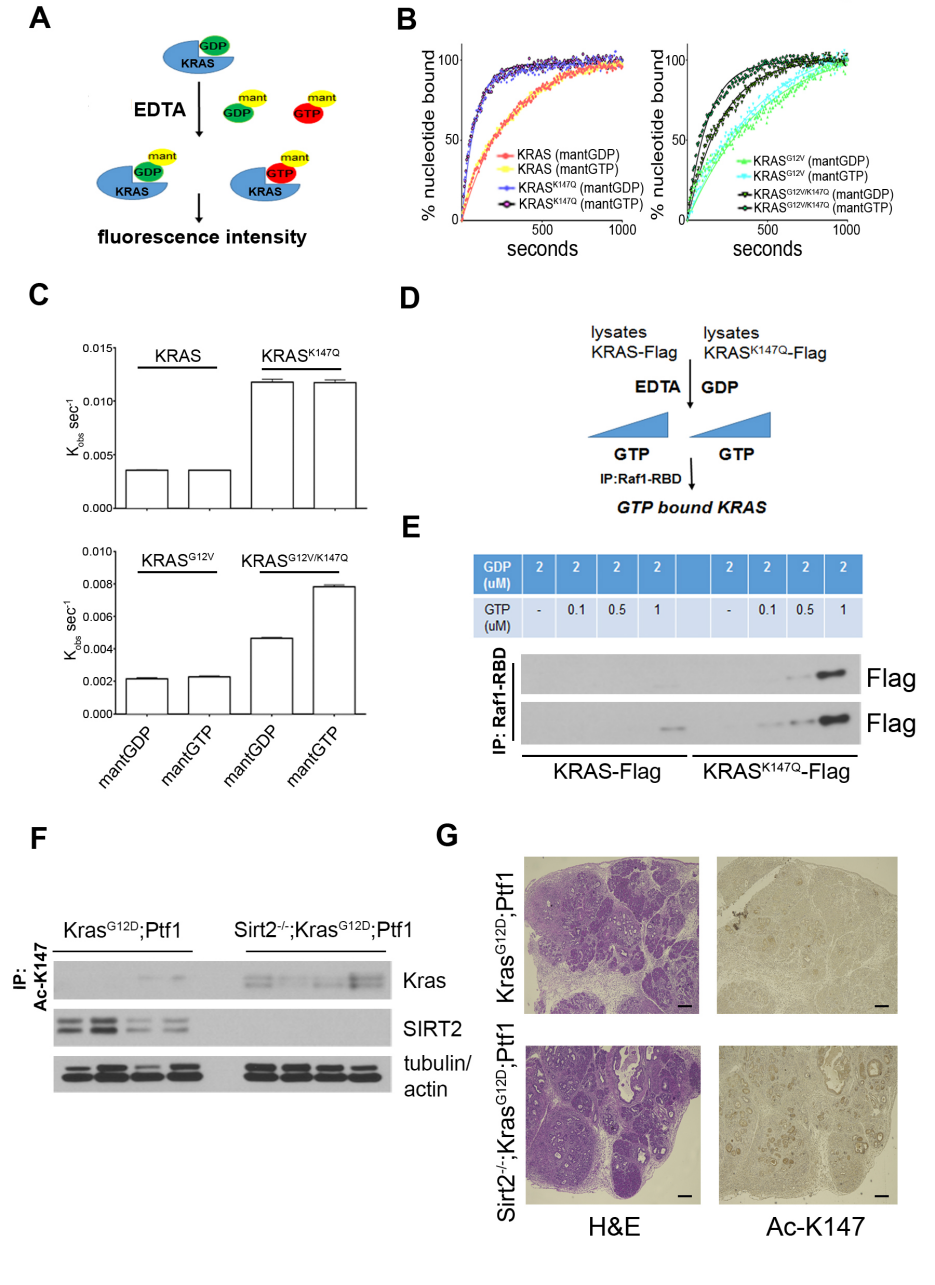
The effect of K147 acetylation on the nucleotide exchange rate along with its detection *in vivo* support the physiological significance of this post-translational modification **A.** Schematic representation of experimental results shown in (B). Purified GDP-loaded KRAS is incubated in the presence of EDTA with fluorescently labeled mant-GDP and mant-GTP. The nucleotide exchange rate is monitored in real-time by measuring the increase in fluorescence intensity after binding of mant-labeled nucleotides to KRAS. **B.** Change in fluorescence over time using the method described in (A) is shown for KRAS and KRAS^K147Q^ (left) as well as KRAS^G12V^ and KRAS^G12V/K147Q^ (right). **C.** First-order rate constants for nucleotide exchange were determined for KRAS and KRAS^K147Q^ (upper) as well as KRAS^G12V^ and KRAS^G12V/K147Q^ (lower). Data represent mean ± SEM. All readings were performed in triplicate. **D.** Schematic representation of experimental results shown in **E**. Extracts from 293T cells expressing *Flag-KRAS* and *Flag-KRAS* were treated with 2 μM GDP, and exchange reactions in the presence of EDTA were carried out after adding excess amounts of GTP. GTP-bound KRAS and KRAS^K147Q^ were finally assessed after immunoprecipitation using Raf1-RBD agarose beads. Immunoprecipitates following the procedure described in (D) were run on a gel, transferred to a PVDF membrane, and immunoblotted using an anti-Flag antibody. Shorter (upper) and longer (lower) exposures of the same membrane are shown. **F.** The pancreata from Kras^G12D^-Ptf1 and Sirt2^-/-^-Kras^G12D^-Ptf1 mice were harvested and analyzed for K147 acetylation by immunoprecipitation using an anti-Ac-K147 antibody followed by western blotting with a KRAS antibody. **G** Pancreas tissue sections from Kras^G12D^-Ptf1 and Sirt2^-/—^Kras^G12D^-Ptf1 mice (*n* = 3) were H&E stained or stained by IHC using an anti Ac-K147 antibody. Representative images are shown (5x). Scale bar 200 μM.

K147 is located within the functionally important G5 box, a critical peptide sequence for the formation of the nucleotide binding site in RAS proteins [18]. Mutation of the adjacent A146 has been shown to activate the transforming potential of KRAS by significantly increasing the nucleotide exchange rate [19]. To shed light on the biochemistry related to the enhanced KRAS activity detected when K147 is mutated to glutamine (resembling the acetylated status), we employed a method using fluorescently labeled GDP and GTP analogues to measure the nucleotide exchange kinetics of KRAS mutants (Figure 5A). The increase in fluorescence intensity when N-methylanthraniloyl (mant)-labeled GDP and GTP bind to purified KRAS, KRAS^K147Q^, KRAS^G12V^ and KRAS^G12V/K147Q^ allows monitoring of nucleotide exchange in real time. Both KRAS^K147Q^ and KRAS^G12V/K147Q^ exhibited an increased rate of nucleotide exchange compared to KRAS and KRAS^G12V^, respectively (Figure 5B, 5C). Given the higher intracellular concentration of GTP compared to GDP, this may explain the increased GTP-bound “active” KRAS detected when K147 is acetylated.

These results were confirmed when a similar approach was followed using extracts from cells expressing Flag-tagged *KRAS* or *KRAS^K147Q^*. In this case, extracts were treated with 2 μM GDP, resulting in loading of both KRAS and KRAS^K147Q^ with GDP, and exchange reactions in the presence of EDTA - catalyzed off- were carried out after adding increasing amounts of GTP. GTP-bound KRAS and KRAS^K147Q^ were then assessed by immunoprecipitation using Raf1-RBD agarose beads (Figure 5D). In accordance with our previous results, KRAS^K147Q^ showed enhanced GDP exchange for GTP compared to KRAS (Figure 5E), suggesting that K147 acetylation may alter the nucleotide exchange kinetics favoring the active KRAS state.

### K147 acetylation is a PTM that can be detected in tissues

We previously observed increased KRAS acetylation levels in tissues when *Sirt2* is lost (Figure 2E-2G). However, acetylation was detected in those experiments using a general pan Ac-K antibody. We now tested whether K147 is specifically acetylated in *Sirt2^-/-^* tissues using a custom-made polyclonal antibody against acetylated K147 (Ac-K147) of KRAS. The specificity of the antibody against KRAS Ac-K147 was checked *in vitro* via dot blot (Figure S5A) as well in cell culture experiments (Figure S5B-E) where K147 acetylation was detected both after overexpressing *KRAS* and after checking endogenous KRAS. Therefore, after completing the validation process, immunoprecipitation experiments using the anti Ac-K147 antibody in pancreas from both Kras^G12D^-Ptf1 and Sirt2^-/-^-Kras^G12D^-Ptf1 mice revealed that K147 acetylation is a PTM that can be detected *in vivo* (Figure 5F), highlighting the physiological significance of this modification. Then, after checking whether this newly developed antibody is suitable for immunohistochemistry (Figure S5F), pancreas from mice with the genotypes described above were stained. Consistently, we detected increased K147 acetylation in pancreas where *Sirt2* is lost (Figure 5G). Interestingly, besides pancreatic lesions that were stained positively for K147 acetylation, the majority of normal acinar cells in the Sirt2^-/—^KRAS^G12D^-Ptf1 mice that were not yet transformed were stained strongly with the anti Ac-K147 antibody, and this was further associated with phosphorylation of ERK (Figure S5G), suggesting that K147 acetylation may be an early event in KRAS-induced tumorigenesis in the context of *Sirt2* deletion. Under these conditions, enhanced downstream KRAS signaling could contribute to enhanced induction of acinar to ductal metaplasia (ADM), as a result of the direct effect of K147 acetylation on KRAS activity.

## DISCUSSION

Based on the well-established observation that increasing age is the most significant risk factor for tumor development, intense research efforts have focused on the role played by sirtuins as the mechanistic link between aging and tumorigenesis. Regarding SIRT2, we have previously shown that *Sirt2*-deficient mice develop tumors in several tissues, providing the first strong genetic evidence that *SIRT2* may function as a tumor suppressor through its role in regulating the anaphase-promoting complex/cyclosome (APC/C) [5]. Although additional studies further support the tumor-suppressive role of SIRT2 [20], it is worth mentioning that SIRT2 has been shown to exert dual functions where it seems to have oncogenic properties as well [21, 22]. This highlights the complexity of sirtuin biology implying that additional aberrant genetic and/or biochemical events are required for and need to be identified to untangle the role of SIRT2 in tumorigenesis, especially within a tissue-specific and genetic context.

Here, we demonstrate that SIRT2 functions as a tumor suppressor in the context of KRAS-dependent tumorigenesis. Mechanistically, we propose that K147 acetylation may increase KRAS activity and exerts a significant impact on cellular behavior as evidenced by increased proliferation, colony formation ability, anchorage independent growth and, finally, tumor growth rate in cells expressing a *K147Q KRAS* mutant resembling the acetylated state. These results, together with the confirmed detection of K147 acetylation in tissues using our anti-Ac-K147 KRAS antibody, reveal for the first time the role of K147 acetylation *in vivo* as a novel KRAS PTM directed by SIRT2. Sirtuins are deacetylation enzymes and as such they have been found to regulate a plethora of substrates with SIRT2 not being an exemption. Thus, it is expected that additional pathways may contribute either synergistically or independently to KRAS-induced tumorigenesis. In this study we provide evidence to further establish the tumor-permissive phenotype observed in mice lacking *Sirt2*, and we show that K147 KRAS acetylation upon *Sirt2* loss plays a significant role in driving cellular transformation and aberrant growth, at least, in tissues where increased Ras activity has been proven to be a driver for tumorigenesis.

*KRAS* is the most frequently mutated oncogene in human cancer [23], which justifies the intensive efforts made to elucidate regulatory mechanisms, signaling transduction, feedback loops, isoform differences, and heterogeneity regarding the mutational landscape, as well as to exploit vulnerabilities in RAS-related tumors [24]. Here we show that K147 acetylation is a PTM directly affecting KRAS activity rather than protein localization, as commonly happens with other PTMs such as farnesylation [25]. This finding, together with recently published studies showing that both K104 acetylation [12] and K147 mono-ubiquitination [26] regulate KRAS activity, even though it is still unknown whether these PTMs play any role in KRAS tumors *in vivo,* indicates that we are just starting to understand and evaluate the contribution of PTMs to fine-tuning KRAS activity. In this regard, the well-known tumor suppressor p53 showcases how complex this regulatory network of PTMs might be [27]. With regard to KRAS, K147Q, which resembles K147 acetylation, increases the SOS-independent nucleotide exchange rate, whereas K147 mono-ubiquitination doesn't affect intrinsic nucleotide dissociation. Instead it impedes GAP-mediated GTP hydrolysis, resulting in Ras activation [28]. The difference in the biochemistry implies that acetylation and mono-ubiquitination exert their impact on KRAS through different mechanisms. Moreover, if the effect of acetylation was through regulating ubiquitination, it would be expected that K147 acetylation (or K147 mutation) would prevent mono-ubiquitination, resulting in decreased KRAS activity. However this is not supported by our experimental results, which further establishes the independent function of these PTMs. Regarding K104 acetylation, biochemical characterization revealed that K104 acetylation renders KRAS more resistant to SOS-dependent nucleotide exchange [12, 29], even though the effect on KRAS activity hasn't been tested directly. Consistent with previous reports, we could detect K104 acetylation in cells overexpressing *KRAS^G12V^*. However, our mass spectrometry analysis revealed K147 acetylation as a reversible lysine acetylation which is specifically deacetylated by SIRT2. Based on our biochemical, cell behavior and, most importantly, *in vivo* analyses, we show that K147 acetylation may exert a positive effect on KRAS activity (in contrast to the decreased KRAS activity proposed upon K104 acetylation), which results in enhanced KRAS-induced tumorigenesis in mouse models. The opposing effect of the acetylation of these two sites, at least at the biochemical level, indicates the distinct nature of the PTMs, which might be reflected at the molecular and cellular levels with respect to enzymes involved in acetylation/deacetylation as well as physiological conditions under which these PTMs regulate KRAS activity.

Consistent with previous studies showing that K147 is within a conserved sequence critical for the formation of the nucleotide binding site [18], we found a significant increase in the nucleotide exchange rate when K147 was mutated to glutamine, which resembles acetylated K147. Given the higher intracellular concentration of GTP, it can be suggested that acetylation of wild-type KRAS may result in a more frequent auto-activation by spontaneously exchanging GDP for GTP, leading to increased aberrant RAS signaling. This could increase mutant KRAS activity as well, based on the observation that most KRAS mutants retain some intrinsic GTPase activity, despite their insensitivity to GAP-mediated GTP hydrolysis, implying that increased nucleotide exchange could positively regulate KRAS activity. The idea that an increase in nucleotide exchange can act synergistically with decreased GTPase activity is supported by data showing that strongly activating mutations in terms of transforming potential represent a combined effect of reduced GTPase activity and increased exchange [19]. Taking this into account and in the context of a recently proposed classification of different KRAS mutations based on biochemical characterization [30], we could predict that some KRAS mutations, such as G12C, G12D, and G12V, would be affected more by K147 acetylation, based on the undetectable effect on nucleotide exchange and the modest effect on GTP hydrolysis compared to other mutations. Moreover, the finding that KRAS K147 seems to be a specific deacetylation target of SIRT2 raises some novel, yet undiscovered, possibilities regarding the crosstalk between non-genetic and genetic factors in KRAS-induced tumors. Taking into consideration that sirtuins respond to both environmental stress and nutrient availability, whereas their activity is altered with aging, it would be trivial to determine in the future whether the SIRT2-directed acetylation status of K147 can, at least in some part, mediate the effects of non-genetic factors known to affect KRAS-induced tumorigenesis [31], [32], [33] providing an additional layer of regulation.

Taking into account the regulatory role of SIRT2, we present here evidence to further establish the tumor suppressor role of SIRT2 in a KRAS-specific context by proposing that the acetylation status of K147 may direct activity and transformative properties. Both *in vitro* assays and *in vivo* mouse models, as well as data showing that K147 acetylation can be detected *in vivo*, underscores the significance of unraveling the molecular and cellular events related to PTMs which may directly fine-tune KRAS activity. Given the so far unsuccessful attempts to therapeutically target KRAS, stemming from the difficulty of predicting which of the many downstream effector pathways is engaged under specific conditions, the deeper understanding of mechanisms regulating RAS activity itself could fill critical knowledge gaps in the RAS biology field. Furthermore, identification of K147 acetylation as a novel post-translational modification directed by SIRT2 may provide a better understanding of the mechanistic link regarding the crosstalk between non-genetic and genetic factors in KRAS driven tumors. Thus it is reasonable to suggest that conditions known to affect sirtuin activity may further regulate KRAS activity and signal output through K147 acetylation even in the presence of mutant KRAS. In this regard and towards possible therapeutic implications, our *in vivo* experimental results suggest that decreasing KRAS acetylation through increased SIRT2 activity could be an approach to limiting tumorigenic potential of KRAS-driven tumors.

## MATERIALS AND METHODS

### Mice

A description of all mice used in this study, as well as details regarding the generation of the Sirt2^fl/fl^ mice, can be found in the Supplementary Materials and Methods section. Mice were housed, fed and treated in accordance with the guidelines approved by the Northwestern University IACUC.

### Histology/immunohistochemistry

Lung and pancreas tissues were harvested and fixed with 10% formalin. Tissues were paraffin embedded, and 4 μm sections were cut and stained with hematoxylin and eosin (H&E) in the Mouse Histology & Phenotyping Laboratory (MHPL) at Northwestern University. Details regarding the protocol for immuhostochemistry can be found in the Supplementary Materials and Methods.

### Cell culture

NIH/3T3 (ATCC^®^ CRL­1658™), 293T (ATCC^®^ CRL-3216^™^) and HCT116 (ATCC^®^ CCL-247^™^) cells were maintained in DMEM medium supplemented with 10% fetal bovine serum (FBS) with antibiotics at 37°C in a humidified atmosphere containing 5% CO_2_ and 95% O_2_. Cell lines were authenticated using CellCheck by IDEXX Bioresearch, and tested for mycoplasma using PlasmoTest™ - Mycoplasma Detection Kit (InvivoGen, Inc). Hras^-/-^;Nras^-/-^;Kras^lox/lox^;RERT^ert/ert^ MEFs were kindly provided by Mariano Barbacid (Spanish National Cancer Research Centre) and were maintained as described previously [17]. Immortalized *Sirt2^+/+^* and *Sirt2^-/-^* MEFs were made as described previously [5]. Cell lines were cultured for less than 20 passages and frozen down from the first 3 passages for further experiments.

### *In vivo* BrdU incorporation

For i*n vivo* BrdU labeling, mice were injected intraperitoneally with 50 mg/kg of BrdU in PBS, pH 7.6, and were sacrificed 1.5 h later. Pancreata were isolated and fixed in 10% formalin, embedded in paraffin, and processed by routine procedures. BrdU incorporation was detected by immunohistochemistry using BrdU antibody (Sigma) according to the manufacturer's instructions.

### RAS activity

GTP-bound “active” KRAS was determined by using the Thermo Scientific Active Ras Pull-Down and Detection Kit (Thermo Scientific) according to the manufacturer's instructions. A GST-fusion protein of the RAS-binding domain (RBD) of Raf1 along with glutathione agarose resin is used to specifically pull down active RAS.

### Intranasal infection

After anesthetization of *Kras^G12D^* and *Sirt2^-/-^;Kras^G12D^* mice using isofluorane, replication-deficient adenovirus expressing Cre recombinase (adenoCRE; Gene Transfer Vector Core, University of Iowa) was administered intranasally. A detailed description of the protocol can be found in the Supplementary materials and methods

### Immunoprecipitation

Cells or tissue samples were lysed using immunoprecipitation (IP) buffer (25mM Tris-HCl pH 7.5, 150 mM NaCl, 1 mM EDTA, 0.1% NP-40, and 5% glycerol). After protein quantification using the Bradford assay, cell extracts (500 μg - 1 mg total protein) were incubated overnight with appropriate antibodies followed by incubation with protein A or G agarose beads for 4 h at 4 °C. After washing five times with IP buffer, immunocomplexes were resolved using SDS-PAGE and analyzed by western blotting. For immunoprecipitation the antibodies mentioned below were used: Ras (Thermo Scientific), KRAS (Proteintech), Ac-K (Immunechem), HA (Sigma), and Ac-KRAS-K147 (Eurogentec).

### Cell proliferation

For measuring cell proliferation, the MTT- [3-(4,5-dimethylthiazol-2-yl)-2,5-diphenyltetrazolium bromide] proliferation assay, cell number counting and colony formation ability were determined using the assays described in Supplementary Materials and Methods.

### Cellular transformation

Transformation ability of NIH3T3 cells was assessed by checking colonies formed by confluent cells as well as by observing anchorage-independent growth in soft agar. A detailed description of the assays used can be found in the Supplementary Materials and Methods.

### Tumor growth analysis

NIH3T3 cells (1 × 10^6^) or MEFs (2 × 10^6^) were subcutaneously injected in each flank of 4-6 week-old male nude athymic mice (nu/nu) (Jackson Laboratory) in 200 μL PBS. Tumor sizes were determined every 1-2 days (NIH3T3) or 3-4 days (MEFs) by measuring the length (*l*) and the width (*w*) of each tumor with an electronic caliper. Tumor volume (V) was calculated using the formula V = *lw^2^*/2. Mice were housed, fed, and treated in accordance with the guidelines approved by the Northwestern University IACUC.

### Nucleotide exchange assay

Each purified KRAS protein was exchanged in 20 mM Tris pH 7.5, 150 mM NaCl, 5 mM b-mercaptoethanol using PD10 desalting columns (GE Healtcare). Protein samples were, next, incubated with 5 mM GDP (50-fold molar excess) in the presence of 20 mM EDTA. For the nucleotide exchange assay, 2 μM GDP-loaded KRAS and 4 μM mant-GTP or mant-GDP were mixed in the assay buffer (20 mM Tris pH 7.5, 150 mM NaCl, 5 mM β-mercaptoethanol, 10 mM MgCl_2_, 20 mM EDTA) and dispensed into a 384-well plate. Fluorescence was measured every 4 s for 15 min at excitation/emission set to 360 nm/440 nm in a Spectramax M3 plate reader (MolecularDevice). Data were exported and analyzed using Graphpad Prism (GraphPad Software, Inc., La Jolla, CA). All readings were performed in triplicate.

### Statistical analysis

Statistical significances were determined by comparing means of different groups using unpaired *t* test or two-way ANOVA followed by post-tests. Graph Pad Prism 6 (Graphpad Software Inc. La Jolla, CA) was used for statistical analysis.

## SUPPLEMENTARY FIGURES


