# Supplementary material for: SIRT2 deletion enhances KRAS-induced tumorigenesis in vivo by regulating K147 acetylation status
Source: Oncotarget. 2016 Sep 13;7(49):80336–49. doi: 10.18632/oncotarget.12015 (PMC5340253; doi:10.18632/oncotarget.12015)
Supplement: Supplementary file 1 [file oncotarget-07-80336-s001.pdf]

SIRT2 deletion enhances KRAS-induced tumorigenesis *in vivo* by regulating K147 acetylation status

Formatted: Left: 1", Right: 1", Top: 1", Bottom: 1"

Supplementary Material

Mice

Sirt2 knockout mice (1) were crossed with the well-established *LSL-Kras*^{G12D} knock-in mouse model (2) (provided by Dr. Harold L. Moses, Vanderbilt University) to generate *Sirt2*$^{+/+}$*;LSL-Kras*^{G12D} and *Sirt2*$^{-/-}$*;LSL-Kras*^{G12D} mice. These mice were next crossed with the *Ptf1*^{Cre} driver line (3) (provided by Dr. Harold L. Moses, Vanderbilt University) to direct recombination in pancreas and generate *Sirt2*$^{+/+}$*;LSL-Kras*^{G12D}*;Ptf1*^{Cre} and *Sirt2*$^{-/-}$*;LSL-Kras*^{G12D}*;Ptf1*^{Cre} mice. Alternatively, the *Sirt2*$^{+/+}$*;LSL-Kras*^{G12D} and *Sirt2*$^{-/-}$*;LSL-Kras*^{G12D} mice were infected intranasally with an adenoCRE virus to direct recombination of the *LSL-Kras*^{G12D} allele and initiate expression of *Kras*^{G12D} in the lungs (4). To generate *Sirt2* conditional knockout mice, embryonic stem (ES) cells were transfected with a targeting vector (containing floxed exons 5-8 of the *Sirt2* gene as well as a floxed neo gene) by electroporation and selected using G418. ES clones were analyzed by both Southern blotting and PCR for cells with the targeted mutation. Next, cells were microinjected into blastocysts from C57BL/6 mice, and chimeric males were bred to C57BL/6 females to obtain germ-line transmission following standard procedures. Offspring carrying a loxP-neo-loxP cassette were crossed with *Ella-Cre* transgenic mice to delete the neo gene and generate *Sirt2* floxed (*Sirt2*$^{fl/fl}$) mice with loxP sites flanking exons 5-8 of the *Sirt2* gene. Mice were genotyped using tail DNA with the following primers: **(a)** 5’-GCC TTA GCT ACA TAG AAG GC-3’, **(b)** 5’-GAA TGA CCT ACA ATG GGC CA-3’, and **(c)** 5’-GTG TAG CCC TGG CTC TTC TA-3’. These mice were crossed with the *LSL-Kras*^{G12D} and *Ptf1*^{Cre} mice in order to generate *Sirt2*$^{fl/fl}$*;LSL-Kras*^{G12D}*;Ptf1*^{Cre} mice where exons 5-8 of the *Sirt2* gene

are deleted and *Kras*^{G12D} is expressed in the pancreas simultaneously. Mice were housed, fed, and treated in accordance with the guidelines approved by the Northwestern University IACUC.

Immunohistochemistry

For immunohistochemistry (IHC), sections were stained using the protocol described below. Briefly, slides were heated via pressure cooker in DAKO retrieval buffer, and endogenous peroxidases quenched in 3% hydrogen peroxide in methanol for 30 min. Tissues were blocked with 5% BSA in PBS for 30 min and exposed to primary antibodies against BrdU (Millipore), CK19 (DSHB, University of Iowa), Ac-KRAS-K147 (Eurogentec), pERK (Abcam and Cell Signaling), Ki67 (Abcam), thyroid transcription factor 1 (Abcam), and SIRT2 (Proteintech) at 1:100-1:200 overnight at 4 °C. Slides were developed using DAKO secondary antibodies and DAB substrate/buffer. Trichrome staining was performed per the manufacturer's instructions (Sigma Aldrich).

Plasmids/transfection/infection

All plasmids for overexpressing SIRT2 have been described previously (1). Flag-tagged *KRAS* and *KRAS*^{G12V} constructs in pCMV2 vector were kindly provided by Lewis C. Cantley (Weil Cornell Medical College). HA-tagged *KRAS* and *KRAS*^{G12V} constructs in pGCN vector were provided by Mark R. Philips (New York University). Site-directed mutagenesis to make K147 acetylation mutants (K147R and K147Q) as well as subcloning to a pCDH-GFP-puro vector (SBI, Systems Biosciences) were performed by Bioinnovatise. For transient overexpression, 293T cells were transfected using polyethylenimine (PEI) at a ratio of 3 μL PEI/μg DNA. For stable overexpression, 293T cells were used to produce viral particles to infect cells (5). All cells

were selected after treating with puromycin (Cellgro) at 2 µg/mL or G418 (Cellgro) at 200 µg/mL for 2 weeks. For knocking down mouse *Kras*, human *SIRT2* and mouse *Sirt2*, target sequences (*Kras*: 5'- GGATTTGGTGCATGCAGTTGA-3', human *SIRT2*: 5'-AAGTAGTGACAGATGGTTGGC-3' and mouse *Sirt2*: 5'-TTCCAGCGTGTCTATGTTCTG-3') subcloned into a pLKO.1-puro vector (Dharmacon), were used to produce viral particles, followed by cell infection, and selection as described earlier. For siRNA experiments, control siRNA (sc-37007) and mouse siSIRT2 (sc-40989) were bought from Santa Cruz Biotechnology.

Cell treatments

To stimulate KRAS signaling, cells were first starved for 24 h in 0.1% FBS medium followed by treatment with EGF (100 ng/mL) (Sigma). To inhibit the activity of endogenous sirtuins, cells were treated with 2 µM nicotinamide (NAM) (Sigma) for 12 h before lysing the cells. To specifically inhibit SIRT2, cells were treated with 5 µM AGK2 (Santa Cruz) for 12 h before cell lysis.

Intranasal infection

AdenoCRE (2.5×10^7 particles, Gene Transfer Vector Core, University of Iowa) was first mixed with Minimum Essential Medium (MEM) and 2 M $CaCl_2$ (final concentration 10 mM) in a final volume of 125 µL/mouse infected. Using a protein gel loading tip, half of the virus mixture (62.5 µL) was administered by placing the pipette tip at the opening of one nostril. The virus solution was slowly expelled from the tip while the mouse inhaled the drop that was forming. Mice were left to recover for 10-15 minutes. After breathing returned to normal, the procedure was repeated with the remaining 62.5 µL of the virus mixture. Mice were monitored for up to 6

months. All procedures were performed in accordance with approved Northwestern University IACUC protocol.

Western blotting

Cells or tissue samples were lysed using buffer A (25 mM Tris-HCl pH 7.5, 150 mM NaCl, 1 mM EDTA, 0.5% NP-40, and 5% glycerol), followed by sonication and centrifugation at 14,000 rpm for 25 min at 4 ^{o}C. After protein quantification using the Bradford assay, equal amounts of protein (20-40 µg) were mixed with 2× Laemmli lysis buffer, boiled for 5 min, separated on a denaturing SDS-polyacrylamide gel, and transferred to a PVDF membrane. The membrane was blocked in 5% milk/PBS/0.05% Tween for 1 h and incubated with antibodies against pERK, ERK (Cell Signaling), Ras (Thermo Scientific), KRAS (Proteintech), tubulin, actin, Flag, HA (Sigma), SIRT2 (Sigma and Proteintech), Ac-K (Millipore), and Ac-KRAS-K147 (Eurogentec). Membranes were washed with PBS/0.05% Tween and then incubated with HRP-conjugated secondary antibody (Santa Cruz Biotech, Santa Cruz, CA) at room temperature for 1 h. Protein banding was analyzed by an enhanced chemiluminiscence method (Amersham Biosciences, Piscataway, NJ) and resolved on x-ray film (Eastman-Kodak, Rochester, NY) per the manufacturer's specifications. Quantification of western blot bands was performed using Gel Analyzer software.

Cell proliferation

For measuring proliferation of cells, three different methods were used. For the MTT- [3-(4,5-dimethylthiazol-2-yl)-2,5-diphenyltetrazolium bromide] proliferation assay, 5x10^{4} cells/well were plated in 24-well plates. At different time points (0-6 days), MTT solution (BosterBio) was

added to the cells and after incubation for another 2 h, the resultant formazan crystals were dissolved in dimethyl sulfoxide (100 µL) and the absorbance intensity measured using an xMark™ Microplate Absorbance Spectrophotometer (Bio-RAD, USA) at 540 nm. All experiments were performed in triplicate, and the proliferation rate was expressed as a percentage relative to the cell absorbance at day 0. For counting the actual cell number, $5x10^4$ cells/well were plated in 24-well plates. At different time points (0-6 days), cell proliferation was estimated after counting cell numbers using the TC20™ Automated Cell Counter (Bio-RAD, USA) following the manufacturer's instructions. For colony forming ability, 100, 250, 400, 500 or 1000 cells per well were plated onto 6-well tissue culture plates in appropriate medium for each cell line used in this assay. After 2 weeks, colonies were stained with crystal violet and counted.

Cellular transformation

Transformation ability of NIH3T3 cells was assessed by checking colonies formed by confluent cells. A total of 5×10^5 cells/well were seeded in 6-well tissue culture plates in complete medium which was replaced every 3-4 days. Fourteen days post-confluence, the cells were fixed and stained with crystal violet (Sigma), and the number of colonies composed of >50 cells was determined. All assays were performed three times. The mean ± SD was calculated from three independent experiments. Cellular transformation was also assessed by observing anchorage-independent growth using the soft agar assay. Briefly, 10^4 cells/well were seeded into 0.35% low melting point agarose (Lonza) on top of a 0.5% agarose layer in 6-well plates. After 3 weeks, colonies were fixed in methanol and stained with 0.1% crystal violet. For quantification, crystal

violet-positive colonies were counted. All assays were performed three times. The mean ± SD was calculated from three independent experiments.

Deacetylation assays

Both in vitro and in vivo deacetylation assays were performed as described previously (5).

Dot blot assay

Samples (2 µL containing 10 ng – 0.5 µg protein) were spotted onto a nitrocellulose membrane at the center of the grid using a narrow-mouth pipette tip. Membrane was left to dry followed by blocking of non-specific sites after incubation with 5% milk in Tris-Buffered Saline/Tween 20 (TBST) for 0.5-1 h at room temperature. Next, the membrane was incubated with the primary antibody diluted in 1% milk in TBST for 30 min at room temperature. After washing three times with TBST (3 x 5 min), the membrane was incubated for 30 min at room temperature with a secondary antibody conjugated to HRP. Finally, membranes were exposed to x-ray films following incubation with a chemiluminiscence reagent.

Custom anti-Ac-K147 antibody development

The anti-Ac-K147 antibody was generated by Eurogentec following the Speedy 28-day protocol. For immunization of rabbits, a peptide carrying acetylated K147 (N-IETSAK(Ac)TRQGVD-C) as well as a control non-acetylated peptide were synthesized and attached to keyhole limpet hemocyaninand (KLH) carrier. After purification, the raised antibody, together with the synthesized peptides used for immunization, was shipped to our laboratory where they were used in additional validation experiments.

GDP/GTP exchange

Cells were lysed following the same procedure as described for the Ras activity assay. 2 mg of cell lysate for each condition was used. For 2 mL total lysate, 40 µL 0.5 M EDTA pH 8.0 was added (for a final concentration of 10mM) followed by vortexing. Then 40 µL 100 mM GDP was added (for a final concentration of 2 mM). Sample was vortexed again and the mixture was incubated at 30°C for 15 minutes with constant agitation. Each 2 mL sample was split in 4 followed by addition of different amounts of GTPγS (10 mM) to the mixture to reach a final concentration of 0, 0.1, 0.5 and 1 mM. After incubating at 30°C for 15 minutes, the exchange reaction was terminated by placing the samples on ice, adding 32 µL of 1M MgCl2 (for a final concentration of 60mM) and vortexing. GTP-bound "active" KRAS was determined by using the Thermo Scientific Active Ras Pull-Down and Detection Kit (Thermo Scientific) according to the manufacturer's instructions.

Mass spectrometry

$KRAS^{G12V}$ was purified from 293T cells expressing either *Flag-KRAS*^{G12V} alone or *Flag-KRAS*^{G12V} and *SIRT2*. For purification, an anti-Flag antibody covalently attached to agarose (ANTI-FLAG® M2 affinity gel, Sigma) was used to immunoprecipitate $KRAS^{G12V}$, followed by elution with excess free Flag peptide according to the procedure described in (6). Eluted samples were run on a 4-20% polyacrylamide gel, followed by silver staining (ThermoFisher Scientific). Protein bands were cut and sent to the MSRC Proteomics Laboratory at Vanderbilt University for mass spectrometry analysis as described before (6).

Cloning, expression, and purification of KRAS proteins

KRAS, $KRAS^{K147Q}$, $KRAS^{G12V}$, and $KRAS^{G12V/K147Q}$ gBlocks® gene fragments (IDT) were cloned into pMCSG7 vector. Each plasmid was digested with SspI restriction enzymes and the linearized vector was gel-purified. Ten μL of Gibson assembly® master mix (New England Biolabs), 1 pmol pMCSG7 linearized vector, and 3 pmol of each gBlock product were incubated for 1 h at 50 °C. One μL of the Gibson assembly® reaction mix was transformed into TOP10 electro-competent cells (Sigma). All the sequences were validated by DNA sequencing. To improve expression and solubility of the KRAS proteins, the catalytic domain of each KRAS construct (residues 1-169) was amplified from purified pMSCG7, using the following primers: FWD 5'-CTTTAAGAAGGAGTCTCTCCCATGACTGAATATAAACTTGTGGTAGTTGG-3' and REV 5'-CCGCTAATGCTGCCCCCTTTTCTTTATGTTTTCGAATTTCTCGAACTAATG-3'. All PCR products were gel-purified and inserted into pMCSG58-CPD_{Vc} vector. pMCSG58-CPD_{Vc} vector comprises the C-terminal domain of the *Vibrio cholerae* MARTX toxin cysteine protease domain (CPD) followed by a 6x His-tag. The addition of inositol hexakisphosphate (InsP6) triggers the CPD autoprocessing, releasing the target protein in solution without the 6His-tag. pMCSG58-CPD_{Vc} was digested with SmaI and gel-purified. The Gibson assembly® cloning method was used to insert each PCR product into pMCSG58-CPD_{Vc} linearized vector as described above. Plasmid insert sequences were confirmed to be accurate by DNA sequencing, and each plasmid was transformed into *E. coli* BL21(DE3)/MAGIC cells. Cultures were grown in Terrific Broth supplemented with 100 µg/mL ampicillin and 30 µg/mL kanamycin at 37 °C until OD_{600} = 0.8 and then induced with 1 mM isopropyl-b-D-thiogalactoside at 25 °C for ≈18 h. Bacteria were

harvested by centrifugation, re-suspended in buffer A1 (50 mM Tris pH 8.3, 500 mM NaCl, 10 mM $MgCl_2$, 0.1% Triton X-100, 5 mM β-mercaptoethanol), and lysed by sonication. After centrifugation at 30,000xg for 30 min, the soluble lysates were loaded onto a 5-mL Ni-NTA HisTrap column using the ÄKTA protein purification system (GE Healthcare). The column was washed with buffer B1 (10 mM Tris pH 7.5, 500 mM NaCl, 10 mM $MgCl_2$, 50 mM imidazole) followed by elution in the same buffer with 500 mM imidazole (buffer C1). Proteins were further purified by size-exclusion chromatography (Superdex 200 (26/60), GE Healthcare) in buffer D1 (10 mM Tris-HCl pH 7.5, 500 mM NaCl, 10 mM $MgCl_2$, 5 mM β-mercaptoethanol). $KRAS_{1\text{-}169}$-CPD fusion proteins were incubated with 100 µM InsP6 (Sigma) for 1 h at 25 °C under gentle agitation. Protein samples treated with Insp6 were loaded again onto a Ni-NTA HisTrap (GE Healthcare), and the flow-through fractions containing $KRAS_{1\text{-}169}$ proteins were collected. Purified proteins were analyzed by SDS-PAGE.

References

1. Kim, H. S., Vassilopoulos, A., Wang, R. H., Lahusen, T., Xiao, Z., Xu, X., Li, C., Veenstra, T. D., Li, B., Yu, H., Ji, J., Wang, X. W., Park, S. H., Cha, Y. I., Gius, D., and Deng, C. X. (2011) SIRT2 maintains genome integrity and suppresses tumorigenesis through regulating APC/C activity. *Cancer cell* **20**, 487-499
2. Hingorani, S. R., Petricoin, E. F., Maitra, A., Rajapakse, V., King, C., Jacobetz, M. A., Ross, S., Conrads, T. P., Veenstra, T. D., Hitt, B. A., Kawaguchi, Y., Johann, D., Liotta, L. A., Crawford, H. C., Putt, M. E., Jacks, T., Wright, C. V., Hruban, R. H., Lowy, A. M., and Tuveson, D. A. (2003) Preinvasive and invasive ductal pancreatic cancer and its early detection in the mouse. *Cancer cell* **4**, 437-450
3. Magnuson, M. A., and Osipovich, A. B. (2013) Pancreas-specific Cre driver lines and considerations for their prudent use. *Cell metabolism* **18**, 9-20
4. DuPage, M., Dooley, A. L., and Jacks, T. (2009) Conditional mouse lung cancer models using adenoviral or lentiviral delivery of Cre recombinase. *Nature protocols* **4**, 1064-1072
5. Song, H. Y., Park, S. H., Kang, H. J., and Vassilopoulos, A. (2016) Deacetylation Assays to Unravel the Interplay between Sirtuins (SIRT2) and Specific Protein-substrates. *Journal of visualized experiments : JoVE*
6. Vassilopoulos, A., Pennington, J. D., Andresson, T., Rees, D. M., Bosley, A. D., Fearnley, I. M., Ham, A., Flynn, C. R., Hill, S., Rose, K. L., Kim, H. S., Deng, C. X., Walker, J. E., and Gius, D. (2014) SIRT3 deacetylates ATP synthase F1 complex proteins in response to nutrient- and exercise-induced stress. *Antioxidants & redox signaling* **21**, 551-564

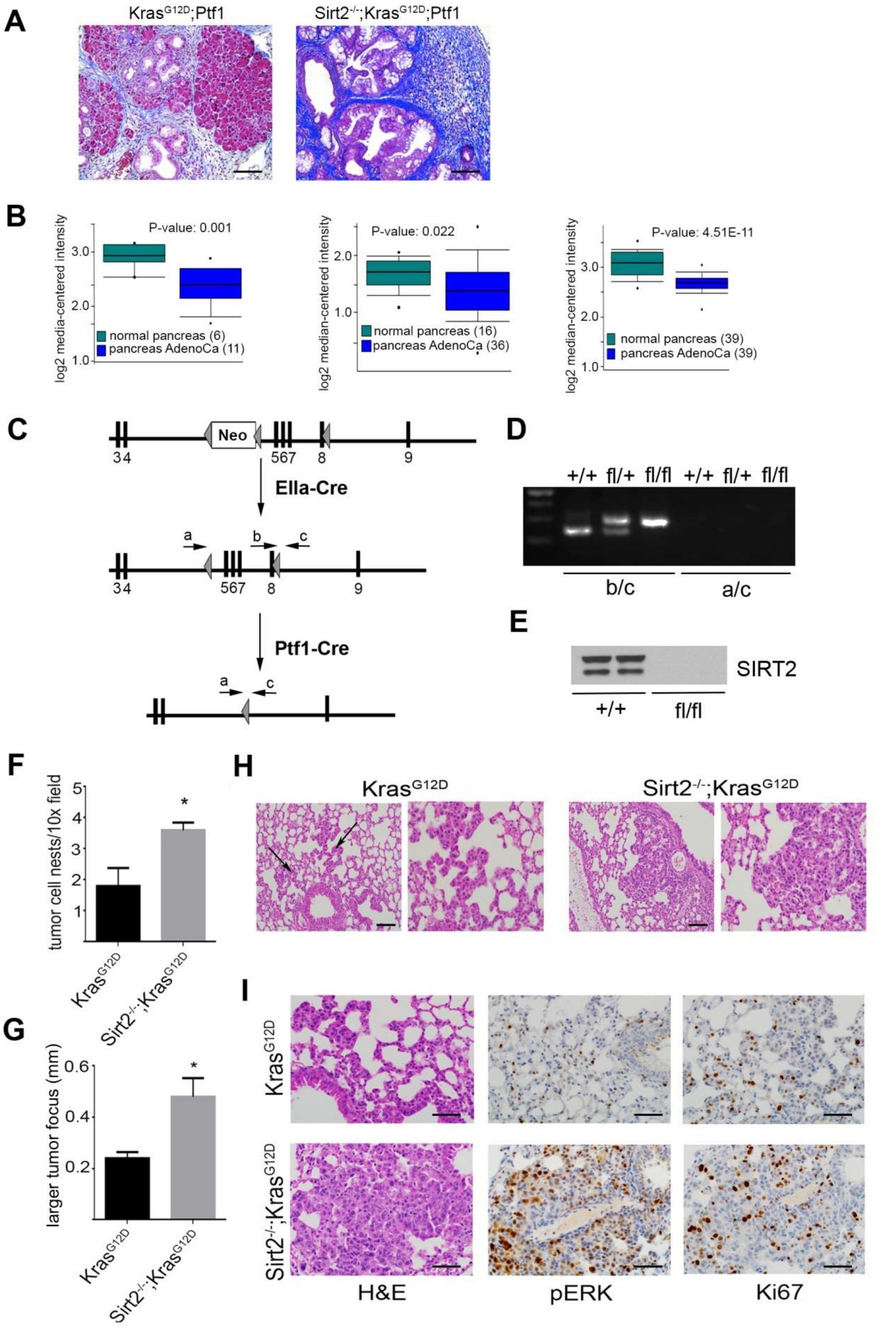
A
KrasG12D;Ptf1
Sirt2-/-;KrasG12D;Ptf1
B
P-value: 0.001
log2 media-centered intensity
normal pancreas (6)
pancreas AdenoCa (11)
P-value: 0.022
log2 median-centered intensity
normal pancreas (16)
pancreas AdenoCa (36)
P-value: 4.51E-11
log2 median-centered intensity
normal pancreas (39)
pancreas AdenoCa (39)
C
Neo
34
567
8
9
EIIa-Cre
a
b
c
Ptf1-Cre
D
+/+ fl/+ fl/fl +/+ fl/+ fl/fl
b/c
a/c
E
SIRT2
+/+
fl/fl
F
tumor cell nests/10x field
KrasG12D
Sirt2-/-;KrasG12D
G
larger tumor focus (mm)
KrasG12D
Sirt2-/-;KrasG12D
H
KrasG12D
Sirt2-/-;KrasG12D
I
KrasG12D
Sirt2-/-;KrasG12D
H&E
pERK
Ki67

Figure S1. *Sirt2* loss enhances KRAS-induced tumorigenesis. (A) The pancreata from $Kras^{G12D}$-Ptf1 and $Sirt2^{-/-}$-$Kras^{G12D}$-Ptf1 mice were stained with a Mallory trichrome stain. Blue staining indicates fibrosis. Representative images are shown (10x). Scale bar 200 μM. **(B)** *SIRT2* expression is decreased in human PDAC samples. The Oncomine genomic microarray database (http://www.oncomine.org) was used to determine *SIRT2* expression in normal pancreas versus PDAC. P values are shown **(C)** Generation of mice carrying pancreas-specific deletion of *Sirt2*. To generate Sirt2 conditional knockout mice, mice carrying a loxP-neo-loxP cassette (upper) were crossed with *Ella-Cre* transgenic mice to delete the neo gene and generate Sirt2 floxed mice with loxP sites flanking exons 5-8 of the *Sirt2* gene (middle). These mice were crossed with *Ptf1-Cre* mice in order to delete exons 5-8 specifically in the pancreas (lower). Pairs of primers (b/c and a/c) used for genotyping are shown. **(D)** PCR-based genotyping of tail DNA using primers b and c to identify wild type (+/+), heterozygous (fl/+), and homozygous (fl/fl) *Sirt2* floxed mice. No *Sirt2* deletion can be detected in tail DNA using primers a and c. **(E)** Western blot using a SIRT2 antibody in pancreata of *Sirt2* floxed mice after crossing with *Ptf1-Cre* mice showing *Sirt2* deletion in pancreas. **(F, G)** The lungs from $Kras^{G12D}$ and $Sirt2^{-/-}$-$Kras^{G12D}$ mice (n=5-8), two months after intranasal administration of adenoCRE, were harvested, fixed, sectioned, and H&E stained. Lung sections from both genotypes were analyzed for: (F) nests of tumor-like cells per 10x field and (G) size of tumors. Data represent mean ± SEM, $*p<0.05$. **(H)** Representative images from each genotype are shown. $Kras^{G12D}$ mice develop mostly atypical adenomatous hyperplasia (AAH) (2 left panels, arrows indicate AAH, 2nd panel shows higher magnification, 20x vs 10x) whereas $Sirt2^{-/-}$-$Kras^{G12D}$ mice develop mostly adenocarcinoma (2 right panels, 2nd panel shows higher magnification, 20x vs 10x). Scale bar 200 μM. **(I)** The $Sirt2^{-/-}$-$Kras^{G12D}$ mice, but not the $Kras^{G12D}$ mice, exhibit increased pERK (middle) and Ki67 (right) staining. The lungs from 3 mice in each group ($Sirt2^{-/-}$-$Kras^{G12D}$ and $Kras^{G12D}$) were harvested and analyzed by IHC staining with anti-pERK and Ki-67 antibodies. Areas of cells with increased staining display a rust color. Representative images are shown (20x). Scale bar 100 μM.

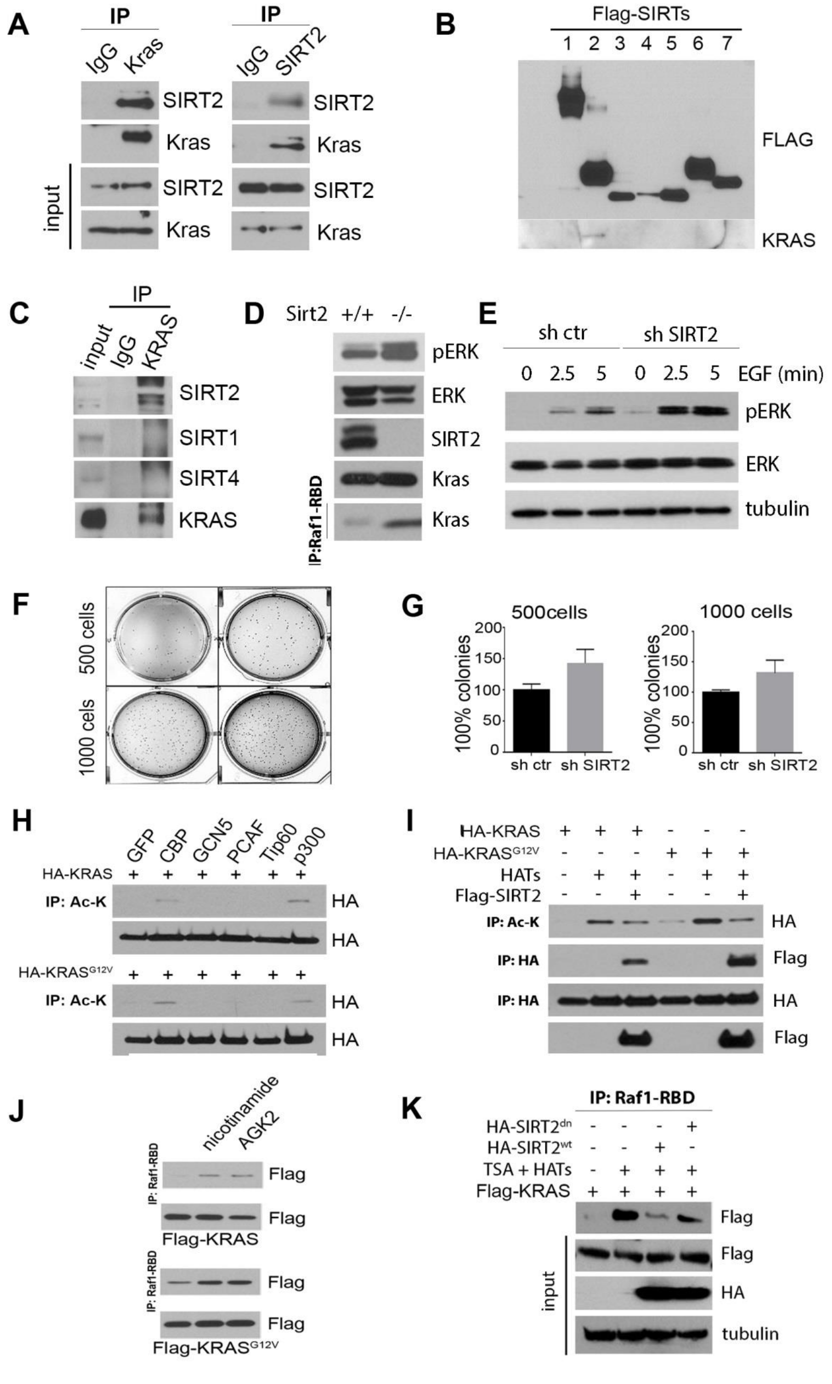

A
IP
IgG
Kras
SIRT2
Kras
input
SIRT2
Kras
IP
IgG
SIRT2
SIRT2
Kras
SIRT2
Kras
B
Flag-SIRTs
1 2 3 4 5 6 7
FLAG
KRAS
C
IP
input
IgG
KRAS
SIRT2
SIRT1
SIRT4
KRAS
D
Sirt2 +/+ -/-
pERK
ERK
SIRT2
Kras
IP:Raf1-RBD
Kras
E
sh ctr
sh SIRT2
0 2.5 5 0 2.5 5 EGF (min)
pERK
ERK
tubulin
F
500 cells
1000 cells
G
500cells
1000 cells
100% colonies
sh ctr
sh SIRT2
H
GFP
CBP
GCN5
PCAF
Tip60
p300
HA-KRAS
IP: Ac-K
HA
HA-KRAS^G12V
I
HA-KRAS
HA-KRAS^G12V
HATs
Flag-SIRT2
IP: Ac-K
IP: HA
HA
Flag
J
nicotinamide
AGK2
IP: Raf1-RBD
Flag
Flag-KRAS
Flag-KRAS^G12V
K
IP: Raf1-RBD
HA-SIRT2^dn
HA-SIRT2^wt
TSA + HATs
Flag-KRAS
Flag
input
HA
tubulin

Figure S2. SIRT2 interacts with KRAS and regulates its activity. (A) Endogenous KRAS and SIRT2 proteins were immunoprecipitated from MEF cell lysates with KRAS (left) and SIRT2 (right) antibodies. Interaction was confirmed by western blotting using anti-SIRT2 and anti-KRAS antibodies, respectively. Endogenous levels of both KRAS and SIRT2 are shown as input. **(B)** Flag-tagged sirtuins (SIRT1-7) were expressed in 293T cells (upper) followed by immunoprecipitation using an anti-Flag antibody. Interacting proteins were eluted after incubation with a Flag peptide and samples were checked for immunoreactivity against KRAS (lower). **(C)** Endogenous KRAS was immunoprecipitated in $Kras^{lox}$ MEFs and interaction with different members of the sirtuin family was checked using specific antibodies as indicated. **(D)** Cell extracts from $Sirt2^{+/+}$ and $Sirt2^{-/-}$ MEFs were analyzed by western blotting using antibodies against pERK, ERK, SIRT2, and KRAS. The same lysates were used to determine the relative amounts of GTP-bound active KRAS through a specific protein interaction with Raf1-RBD. **(E)** HCT116 cells were infected with lenti-*shluc* (sh ctr) or lenti-*shSIRT2* (sh SIRT2), treated with EGF (100 ng/mL) for 2.5 and 5 minutes, and extracts were immunoblotted with antibodies against pERK, ERK, and tubulin. **(F, G)** Totals of 500 and 1,000 HCT116-sh ctr and HCT116-sh SIRT2 cells were plated in soft agar for 21 days (F) and colonies were counted (G). All experiments were done in triplicate, and representative images are shown. **(H)** 293T cells were co-transfected with either *HA-KRAS* (upper) or $HA\text{-}KRAS^{G12D}$ (lower), as well as different HATs. Acetylated levels of both HA-KRAS and HA-KRAS^{G12D} were determined after immunoprecipitation using an anti-Ac-K antibody followed by immunoblotting with an anti-HA antibody. Levels of expressed KRAS and KRAS^{G12V} are shown as input after immunoblotting with an anti-HA antibody. (**I**) 293T cells were co-transfected with either *HA-KRAS* or $HA\text{-}KRAS^{G12V}$, HATs, and wild-type *Flag-SIRT2*. Acetylated levels of both KRAS and KRAS^{G12V} were determined after immunoprecipitation using an anti-Ac-K antibody followed by western blotting using an anti-HA antibody. Interaction of both KRAS and KRAS^{G12V} with SIRT2 was confirmed after immunoprecipitation using an anti-HA antibody followed by western blotting using an anti-Flag antibody, whereas successful immunoprecipitation of both KRAS and KRAS^{G12V} was confirmed by western blotting using an anti-HA antibody. Levels of expressed SIRT2 are shown after immunoblotting using an anti-Flag antibody. **(J)** 293T cells were transfected with either *Flag-KRAS* (upper) or $Flag\text{-}KRAS^{G12V}$ (lower) and cells were subsequently treated with nicotinamide and AGK2. The relative amounts of GTP-bound active Ras were determined through a specific protein interaction with Raf1-RBD. Levels of expressed KRAS and KRAS^{G12V} are shown after immunoblotting using an anti-Flag antibody. **(K)** 293T cells were co-transfected with *Flag-KRAS*, *HATs*, and either $HA\text{-}SIRT2^{wt}$ (wild-type SIRT2) or $HA\text{-}SIRT2^{dn}$ (deacetylation null mutant SIRT2). KRAS activity was determined as described in (J). Levels of expressed KRAS and SIRT2 are shown after immunoblotting using anti-Flag and anti-HA antibodies, respectively. Tubulin is used as loading control.

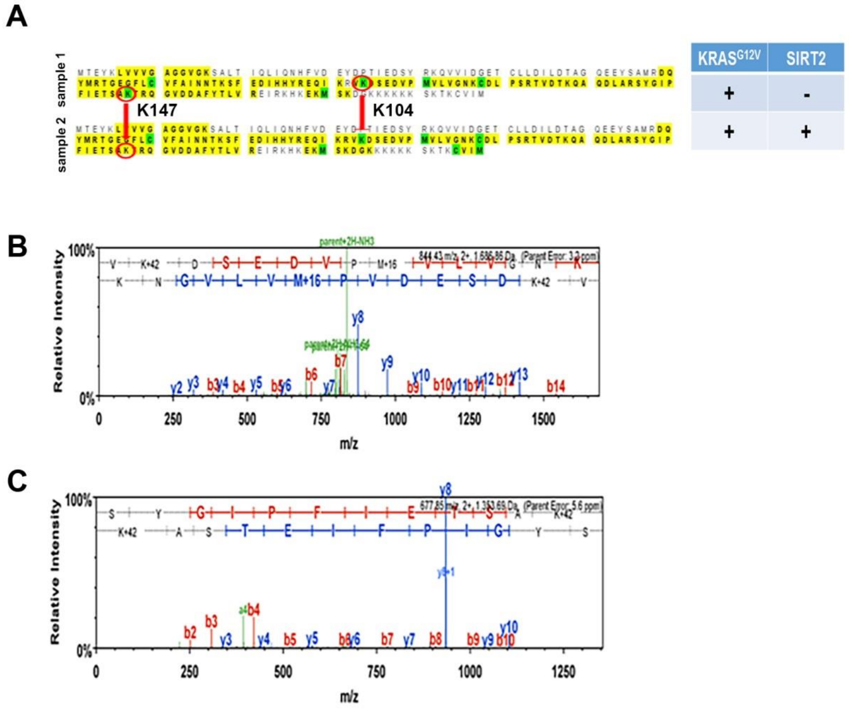

Figure S3. K147 is a SIRT2 deacetylation target. (A) 293T cells were transfected with *Flag-KRAS*^{G12V} in the absence (sample 1) or presence (sample 2) of exogenously expressed *SIRT2*. After immunoprecipitation using an anti-Flag antibody, samples were separated on a gel, and bands corresponding to Flag-tagged KRAS^{G12V} were excised, in-gel trypsin digested, and analyzed by mass spectrometry. Identified peptides are highlighted in yellow, and acetylated lysines are indicated by red circles. K104 and K147 were found to be acetylated in sample 1, whereas K147 acetylation was not detected in sample 2. **(B, C)** Spectra for both sites found to be acetylated in sample 1 are shown.

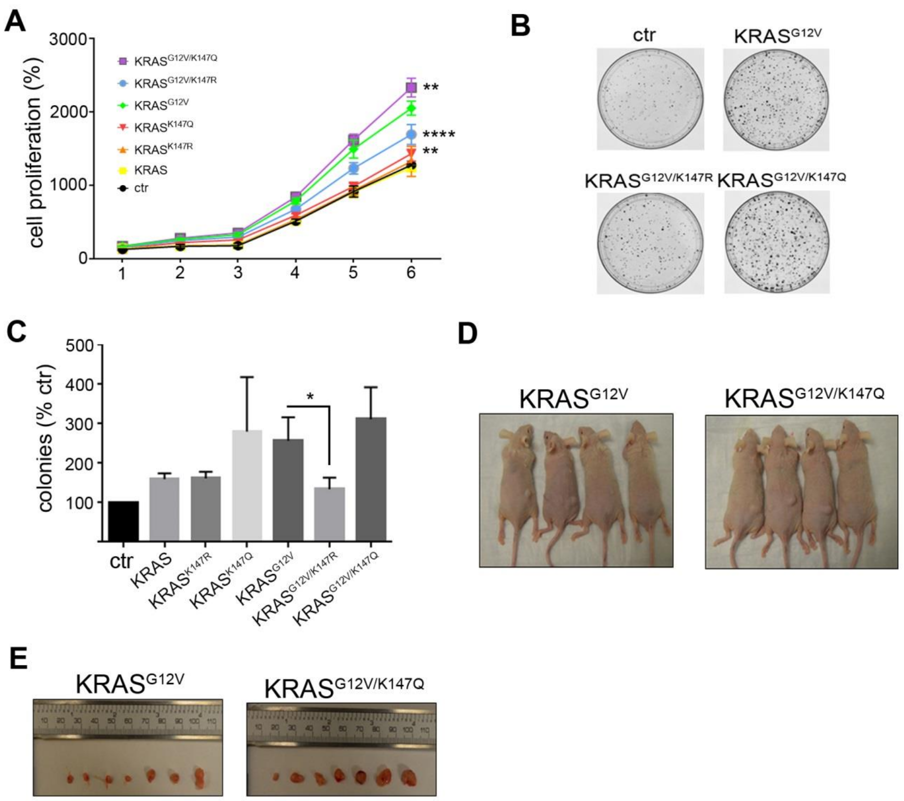

Figure S4. K147 acetylation enhances transformation activity of KRAS and plays a role in regulating guanine nucleotide exchange. (A-C) Cell proliferation in NIH3T3 cells expressing *KRAS*, $KRAS^{147R}$, $KRAS^{147Q}$, $KRAS^{G12V}$, $KRAS^{G12V\text{-}K147R}$ and $KRAS^{G12V\text{-}K147Q}$ after knocking down endogenous *Kras* was checked by measuring the number of cells for 6 consecutive days (A) and by determining the number of colonies formed after 21 days (B, C). For cell proliferation, data represent mean ± SD of three independent experiments, $^{**}p<0.01$ $KRAS^{G12V/K147Q}$ vs $KRAS^{G12V}$ cells, $^{****}p<0.0001$ $KRAS^{G12V/K147R}$ vs $KRAS^{G12V}$ cells, $^{**}p<0.01$ $KRAS^{K147Q}$ vs *KRAS* cells. For number of colonies formed, data represent mean ± SD of three independent experiments, $^{*}p<0.05$ $KRAS^{G12V/K147R}$ vs $KRAS^{G12V}$ cells. **(D)** Representative images of mice bearing the subcutaneous tumors after injecting with $Kras^{lox}$ MEFs infected with lenti-$KRAS^{G12V}$, lenti-$KRAS^{G12V\text{-}K147R}$, and lenti-$KRAS^{G12V\text{-}K147Q}$ followed by treatment with 4HT. **(E)** Representative photos of subcutaneous tumors removed after sacrificing the mice injected with the cells described in (D).

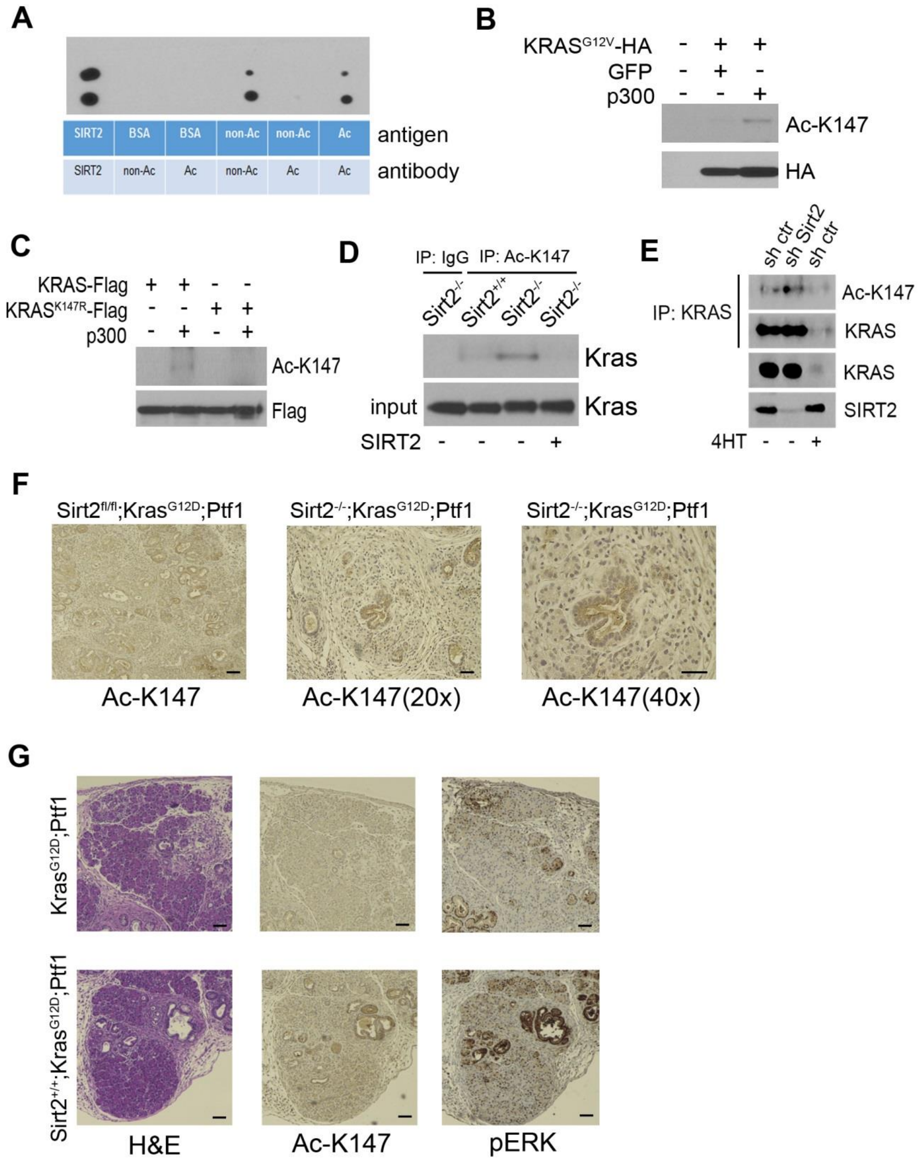

Figure S5. K147 acetylation can be detected *in vivo* by using a KRAS-Ac-K147 antibody. (A) A dot blot assay was performed using different combinations of antigen/antibody: lane 1, SIRT2/SIRT2; lane 2, BSA/non acetylated KRAS (non Ac); lane 3, BSA/acetylated KRAS (Ac); lane 4, non Ac-KRAS peptide/non Ac-KRAS; lane 5, non Ac-KRAS peptide/Ac-KRAS; lane 6, Ac-KRAS peptide/Ac-KRAS. **(B)** Lysates from 293T cells co-transfected with *HA-KRAS^{G12V}* and either GFP (as control) or p300 were immunoblotted with the anti-KRAS-Ac-K147

antibody. Levels of expressed $KRAS^{G12V}$ are shown after immunoblotting using anti-HA antibody. **(C)** Lysates from 293T cells transfected with either *Flag-KRAS* or *Flag-*$KRAS^{K147R}$ in the absence or presence of p300 were immunoblotted with the anti-KRAS-Ac-K147 antibody. **(D)** Lysates from both *Sirt2*$^{+/+}$ and *Sirt2*$^{-/-}$ MEFs were immunoprecipitated using the anti-KRAS-Ac-K147 antibody followed by immunoblotting with a KRAS antibody. *Sirt2*$^{-/-}$ MEF lysates were also immunoprecipitated using rabbit IgG as a negative control (lane 1). **(E)** KRAS was immunoprecipitated in *Kras*^{lox} MEFs followed by western blotting using the anti-Ac-K147 specific antibody. *Sirt2* knockdown efficiency (after *shSIRT2* lentiviral infection) and *Kras* knockdown efficiency (upon 4-HT treatment) was confirmed by immunoblotting using SIRT2 and KRAS antibodies. **(F)** Pancreas tissue sections from $Sirt2^{fl/fl}$-$Kras^{G12D}$-Ptf1 (left, 5x, scale bar 200 μM)) and $Sirt2^{-/-}$-$Kras^{G12D}$-Ptf1 mice were stained by IHC using an anti-KRAS-Ac-K147 antibody. Middle (20x) and right (40x) panels show higher magnification of stained tissues shown in Figure 5G. Scale bar 50 μM. **(G)** Pancreas tissue sections including areas with normal acinar cells from $Kras^{G12D}$-Ptf1 (upper) and $Sirt2^{-/-}$-$Kras^{G12D}$-Ptf1 (lower) mice were H&E stained (left) or stained by IHC using an anti-KRAS-Ac-K147 (middle) and an anti-pERK (right) antibody. Representative images are shown (5x). Scale bar 200 μM.
